# Gravity-dominated Collisions: A Model for the Largest Remnant Masses with Treatment for “Hit and Run” and Density Stratification

**DOI:** 10.3847/1538-4357/ab528d

**Published:** 2020-03-24

**Authors:** Travis S. J. Gabriel, Alan P. Jackson, Erik Asphaug, Andreas Reufer, Martin Jutzi, Willy Benz

**Affiliations:** 1 Arizona State University, 781 E Terrace Mall, ISTB4, Room 795, Tempe, AZ 85287-6004, USA; 2 Centre for Planetary Sciences, University of Toronto, 1265 Military Trail, Toronto, Ontario, M1C 1A4, Canada; 3 Lunar and Planetary Institute, University of Arizona, 1629 E University Boulevard, Tucson, AZ 85721, USA; 4 Physikalisches Institut, Universität Bern, Sidlerstrasse 5, CH-3012, Bern, Switzerland

**Keywords:** Impact phenomena (779), Planetary science (1255), Planet formation (1241), Hydrodynamics (1963), Hydrodynamical simulations (767), Inner planets (797)

## Abstract

We develop empirical relationships for the accretion and erosion of colliding gravity-dominated bodies of various compositions under conditions expected in late-stage solar system formation. These are fast, easily coded relationships based on a large database of smoothed particle hydrodynamics (SPH) simulations of collisions between bodies of different compositions, including those that are water rich. The accuracy of these relations is also comparable to the deviations of results between different SPH codes and initial thermal/rotational conditions. We illustrate the paucity of disruptive collisions between major bodies, as compared to collisions between less massive planetesimals in late-stage planet formation, and thus focus on more probable, low-velocity collisions, though our relations remain relevant to disruptive collisions as well. We also pay particular attention to the transition zone between merging collisions and those where the impactor does not merge with the target, but continues downrange, a “hit-and-run” collision. We find that hit-and-run collisions likely occur more often in density-stratified bodies and across a wider range of impact angles than suggested by the most commonly used analytic approximation. We also identify a possible transitional zone in gravity-dominated collisions where larger bodies may undergo more disruptive collisions when the impact velocity exceeds the sound speed, though understanding this transition warrants further study. Our results are contrary to the commonly assumed invariance of total mass (scale), density structure, and material composition on the largest remnants of giant impacts. We provide an algorithm for adopting our model into *N*-body planet formation simulations, so that the mass of growing planets and debris can be tracked.

## 1. Introduction

Planet-scale collisions gained prominence in the context of terrestrial planet formation in the post-*Apollo* era (e.g., Hartmann & Davis 1975; Wetherill 1985). A large body of subsequent work has led to the widely accepted prediction that the final phases of the growth of small, nongaseous planets is dominated by chaotic collisions between planetary embryos (e.g., Kenyon & Bromley 2006; Raymond et al. 2009; Kokubo & Genda 2010). Giant impacts between planetary embryos are also a key feature within the newly introduced planet formation framework “pebble accretion” (e.g., Morbidelli et al. 2015), which features the swift accretion of planetary embryos from small components. There is substantial evidence for the occurrence of these giant, planetary-scale impacts on terrestrial planets in our own solar system, with such impacts implicated in the origin of the Moon (e.g., Cameron & Ward 1976; Benz et al. 1986; Canup & Asphaug 2001; Canup 2004), the formation of Mercury (e.g., Benz et al. 2007; Asphaug & Reufer 2014), and the genesis of the Martian hemispheric dichotomy/Borealis basin (Wilhelms & Squyres 1984; Marinova et al. 2008, 2011). In the particular case of the Moon, not only do we have evidence in the existence of the Moon itself, but also potentially from signatures left by subsequent collisions between debris released by the impact and the asteroid belt (Bottke et al. 2015). Indeed, the only one of the terrestrial planets for which we do not currently have direct evidence of a giant-impact event is Venus, and it is likely not a coincidence that Venus is the planet about whose surface we know the least. Planetary-scale collisions are by no means limited to the inner regions of the solar system. In the Kuiper Belt, the Pluto–Charon binary system is believed to have formed in an impact much like that which formed our own Moon (e.g., Canup 2005, 2011; Stern et al. 2006). The Haumea collisional family (Brown et al. 2007; Leinhardt et al. 2010) also likely has its origin in a giant impact, and the Saturnian satellites are theorized as remnants of the giant-impact accretion of Titan (e.g., Asphaug & Reufer 2013).

We are also accruing evidence for the occurrence of giant impacts in other planetary systems. The strongest candidate is probably the HD 172555 system, for which the mid-infrared spectrum shows the presence of copious quantities of amorphous silica dust in the terrestrial region of this 12 Myr old A5V star (Lisse et al. 2009). Because amorphous silica is produced by the fast quenching of molten material, this points to the dust being generated by a recent collision at >10 km s^−1^, equivalent to planetary escape velocities, between two massive protoplanets which would produce large volumes of melt (Lisse et al. 2009). Another candidate is the A-star Beta Pictoris. Observations by Telesco et al. (2005) revealed a large brightness asymmetry in the mid-infrared at a projected separation of about 50 au from the star. More recent observations with the Atacama Large Millimetre Array by Dent et al. (2014) reveal colocated asymmetries in the submillimeter continuum and in CO gas. One explanation for the origin of this asymmetry in the Beta Pictoris debris disk is a recent (in the last million years) collision between two icy protoplanets (Dent et al. 2014; Jackson et al. 2014). As members of the Beta Pictoris moving group (29 pc and 19.44 pc distant respectively), HD 172555 and Beta Pictoris are two of the nearest young A stars to the solar system. Taken together, they provide potential nearby analogs of the giant impacts that have been inferred for our own inner and outer solar system.

Alongside these observational considerations, theoretical work has shown that properly accounting for the outcomes of giant impacts during the chaotic phase of terrestrial planet formation is likely essential to accurately model the final planetary system. Earlier *N*-body planet formation simulations such as Chambers (2004) or Kenyon & Bromley (2006) used a perfect-merger model for the outcome of collisions, where two colliding bodies will always result in a single body with mass equal to the sum of the two. In contrast detailed modeling of individual impacts using hydrocode simulations, such as that of Agnor & Asphaug (2004) and Asphaug et al. (2006), showed that the perfect-merger model is not realistic. In particular, hydrocode simulations of off-axis collisions between similarly sized bodies have shown that the class of “hit-and-run” collisions, in which the bodies collide without significant accretion or disruption, is important, especially with regards to the fate of the impactor that continues on a deflected heliocentric orbit. For typical velocities in a dynamical system of self-stirred bodies, hit-and-run collisions appear to happen as frequently as effective mergers (e.g., Agnor & Asphaug 2004). Furthermore, the impactor that deflects downrange can reimpact the same target at timescales ~10^3^–10^5^ yr on average at 1 au, or collide with another body (Emsenhuber & Asphaug 2019). In either case, subsequent collisions may also be hit and run (Emsenhuber & Asphaug 2019), serving as an efficient transport mechanism between growing planetary bodies.

It is clear that incorporating a more realistic accretion model into *N*-body simulations is highly desirable; however, modeling of individual giant impacts shows that they can result in a wide variety of outcomes (e.g., Asphaug 2010). Attempts have been made (Genda et al. 2011) to construct hybrid methods in which an *N*-body simulation spawns a smoothed particle hydrodynamics (SPH) simulation when a collision is detected. The *N*-body simulation is temporarily halted, then resumed once it is provided the output of the SPH simulation. This is exceptionally computationally expensive because the *N*-body code integrates for millions of years (months of wall-clock time), each SPH simulation integrates for hours (days of wall-clock time), and hundreds of such collisions between large bodies will occur in *N*-body models of late-stage planet formation. Thus far, no such scheme has successfully been demonstrated in the literature. With a true hybrid method beyond reach, at least in the near term, efforts have focused instead on characterizing the parameter space of giant-impact outcomes and constructing analytical relationships to link the pre-impact parameters to the post-impact outcomes in models that serve as a surrogate for SPH. Fully data-driven methods are also possible such as interpolation or machine learning (Cambioni et al. 2019), which do not rely on underlying assumptions of human-derived analytical forms. Ideally, the models apply to a large range of conditions and scales (i.e., total colliding mass). The most comprehensive effort to date to conduct a characterization of the outcomes of giant impacts through physically motivated analytical forms (“scaling laws”) is that of Leinhardt & Stewart (2012) and Stewart & Leinhardt (2012), and their model has now begun to be directly incorporated into the next generation of *N*-body models (e.g., Chambers 2013; Leinhardt et al. 2015).

Previous efforts at characterizing the outcomes of giant impacts have drawn on the wealth of existing laboratory-scale impact data and literature for impacts and asteroids, with catastrophic disruption of the target (the impact energy that produces the largest remnant that is half of the total colliding mass) used as the key point of comparison, as disruption is readily achieved in small bodies in the laboratory and in the asteroid belt. We note that there are issues with the definition of catastrophic disruption for off-axis collisions between similar-sized bodies, as we discuss in Section 2. In a dynamical system of self-stirred bodies, such as a population of planetary embryos, impact velocities are at most a few times the escape velocity, which is well below the catastrophic disruption threshold, as we discuss further in Section 2.1. In contrast small bodies embedded within a population of larger bodies, such as asteroids in the present-day solar system, will collide at similar absolute velocities to the larger bodies, and thus at much larger multiples of their escape velocity, such that collisions between the small bodies will be largely disruptive.

In this work, we focus on collisions of planetary embryos whose impact outcomes, namely the mass of remnants, are dominated by gravity. Collisions of this scale are relevant to the collisions between interacting particles in modern *N*-body codes (e.g., Chambers 2013). We begin by providing an overview of the landscape of giant-impact parameters and outcomes, particularly as it applies to terrestrial planet formation, in Section 2. Then we review past efforts at constructing scaling laws used to predict giant-impact outcomes in Section 3. We describe our methodology in Section 4 before presenting our results in Section 5. In Section 6, we compare our results with previous work and discuss the applicability of our empirical relationships to the range of gravity-dominated collisions. A key product of our work is a simple prescription for incorporating giant-impact outcomes into *N*-body codes, which we present in Section 7. Finally, we summarize our conclusions in Section 8.

## 2. The Landscape of Giant Impacts

Unlike in the cratering of planetary surfaces, collisions between similar-sized bodies feature an impactor whose mass is not negligible by definition. Typically, giant impacts do not result in long-lasting surface morphologies resembling classical impact craters, and any surface deformation is often erased within a few gravitational timescales. A possible exception is the Borealis basin impact hypothesis for the origin of the hemispheric dichotomy on Mars (Marinova et al. 2008, 2011). We note, however, that the impactor-to-target mass ratio in that case is small, ~0.01, and thus this basin-forming collision can be thought of as being in the transition between cratering and similar-sized collisions.

A key parameter of giant impacts is the two-body escape velocity of the target and impactor,

(1) $$\nu_{\text{esc}}={\left(2G\frac{M_{\text{tar}}+M_{\text{imp}}}{R_{\text{tar}}+R_{\text{imp}}}\right)}^{\frac{1}{2}},$$

where *M*_tar_ and *R*_tar_ are the mass and radius of the target, respectively, and *M*_imp_ and *R*_imp_ are the mass and radius of the impactor, respectively. Equation (1) is an adjustment from the single-body form, $\nu_{\text{esc}}=\sqrt{2GM_{\text{tar}}/R_{\text{tar}}}$, to account for the comparable mass and size of the impactor with respect to the target. The impactor-to-target mass ratio is defined by

(2) $$\gamma =M_{\text{imp}}/M_{\text{tar}},$$

so 0 < *γ* ⩽ 1 and, depending on the bulk densities of the bodies, it is still possible that *R*_imp_ > *R*_tar_, a scenario simulated in Movshovitz et al. (2016).

Another key parameter is the impact angle, *θ*_imp_, which we define to be 0° for a head-on collision and 90° for a perfectly grazing geometry. The familiar result of Shoemaker (1962) applies equally to giant impacts as it does to cratering projectiles: that the probability distribution of impact angles goes according to *P*(*θ*_imp_) = sin(2*θ*_imp_), which has a modal value of 45°. In collisions of similar-sized bodies, there is not a lot of material overlap between the impactor and the target for angles greater than around 30°, and 75% of collisions occur at angles greater than 30°. So even moderate-velocity giant impacts can be “hit and run” (Asphaug et al. 2006), with a sizable portion of the impactor continuing downrange, a phenomenon first reported in Agnor & Asphaug (2004). As we show later, stratified planets can even hit and run at angles less than 20°.

### 2.1. Paucity of Disruptive Collisions

A common tool used to understand giant-impact outcomes and to develop predictions for the mass of the largest remnant has been the catastrophic disruption threshold. This represents the energy, Q*. at which the mass of the largest remnant is equal to half of the colliding mass, *M*_LR_ = 0.5*M*_tot_ = 0.5(*M*_tar_ + *M*_imp_). In the case where the projectile is much smaller than the target, specific impact energy takes on the familiar form

(3) $$Q=\frac{1}{2}\frac{M_{\text{imp}}\nu_{\text{imp}}^{2}}{M_{\text{tar}}},$$

where *M*_imp_, *M*_tar_, and *ν*_imp_ are the impactor mass, target mass, and impact velocity, respectively. Because the impact velocities of growing planets are governed by the mutual escape velocities of the bodies, giant impacts span a range of impact velocities, which we can compare to disruption velocities (derived from Q*) reported in the literature.

**Figure 1.**
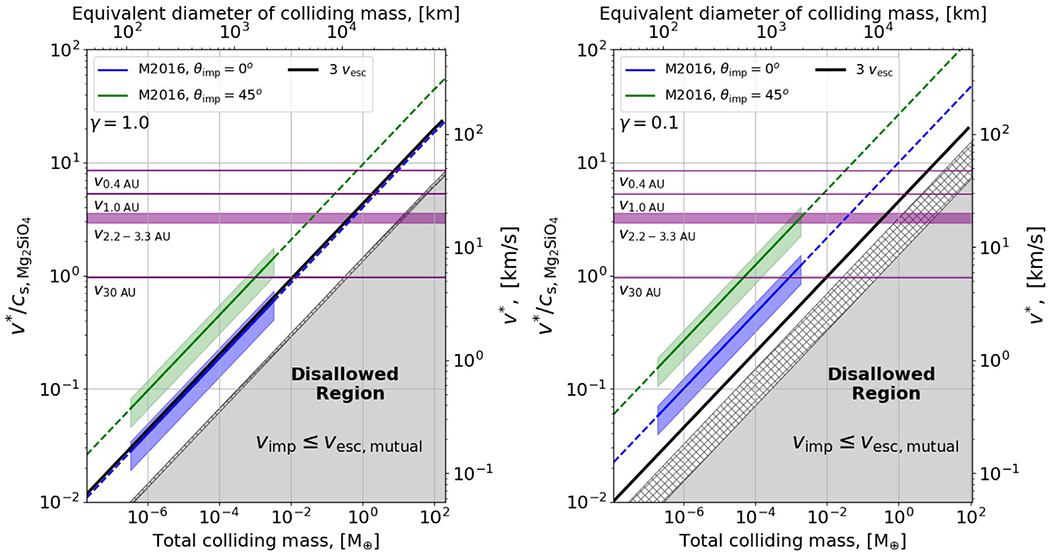
Comparison between collision velocities to disruption thresholds. The blue and green lines represent catastrophic disruption velocities from Movshovitz et al. (2016) for head-on and *θ*_imp_ = 45° collisions, with the shaded bands showing their uncertainties. Velocity in the left-hand *y*-axis is scaled by the sound speed of forsterite at 0 °C and 1 atm (~5.6 km s^−1^; Suzuki et al. 1983). The gray zone represents velocities less than *ν*_esc_, which are disallowed for two-body interactions, and the hatched zone represents impact energy less than half the mutual gravitational binding energy. The purple horizontal lines indicate circular velocities at different radial positions in the solar system. Chambers (2013) finds all embryo–embryo impacts occur below 3*ν*_esc_ (black dotted line) in terrestrial solar system formation. The mutual escape velocity *ν*_esc,mutual_ was computed assuming *ρ*_bulk_ = 3000 kg m^−3^ and an impactor-to-target mass ratio of *γ* = *M*_imp_/*M*_tar_ = 1 (left) and *γ* = 0.1 (right).

In Figure 1, we compare the disruption threshold velocity to impact velocities in which collisions between gravity-dominated bodies will take place, for an impactor-to-target mass ratio of *γ* = 1 on the left and *γ* = 0.1 on the right. The horizontal purple lines mark the circular orbital velocities at roughly the locations of Mercury, Earth, the asteroid belt, and Neptune and thus represent indicative boundaries on the maximum possible impact velocity at these locations.^5^ The blue and green diagonal lines meanwhile show the disruption thresholds from Movshovitz et al. (2016) for head-on and *θ*_imp_ = 45° collisions respectively, both calculated for the appropriate value of *γ*, as a less massive impactor must travel faster to have the same kinetic energy. We can readily see that it is highly implausible for a body larger than ~1 *M*_⊕_ to be disrupted at an orbital distance of 1 au because the maximum impact velocity is lower than the blue and green disruption lines at those masses. In the main belt region, it is nearly impossible for ~1 *M*_⊕_, *γ* = 0.1 collisions to result in disruption because impact energies are strictly in the hatched zone in Figure 1 (right). Larger bodies can be disrupted at smaller orbital distances, but at larger orbital distances, our analysis becomes even more stringent—beyond the orbit of Uranus, even lunar mass bodies (~10^−2^ *M*_⊕_) cannot collide destructively.

At ~30 au, the circular velocity (5.4 km s^−1^) is close to the bulk sound speed of forsterite at 0 °C and 1 atm, ~5.6 km s^−1^ (Suzuki et al. 1983). The sound speed of water is lower at 0 °C and 1 atm: ~1.4 km s^−1^ in the liquid phase (e.g., Smith & Lawson 1954) and ~2.1 km s^−1^ in the solid (Ih) phase (e.g., Vogt et al. 2008), so the onset of shock-producing collisions occurs at lower velocities for outer solar system bodies.^6^ In the absence of damping forces like gas drag, two bodies must always collide at speeds greater than their mutual escape velocity, and thus the gray region, where *ν*_imp_ < *ν*_esc_, is disallowed. The hatched and gray zone together is the region where the impact kinetic energy is less than half the mutual gravitational binding energy, and disruptive outcomes have not been observed in this regime. As such, collisions between equal-sized bodies with *M*_tar_ ≈ 0.2*M*_⊕_ will always be supersonic, as even in the lowest velocity collisions possible (~1*ν*_esc_) the sound speed is exceeded. Note that in regions where *ν*_esc_ exceeds the orbital velocity (purple lines), our analysis implies that collisions are not possible; in this region, the outcome of scattering events is dominated by the ejection of one of the bodies from the system rather than by collisions (e.g., Wyatt et al. 2017).

Alongside the indicative boundaries provided by the orbital velocity and the escape velocity we also show black diagonal lines at 3*ν*_esc_. These correspond to the results of terrestrial planet formation simulations from Chambers (2013) that show all embryo–embryo impacts^7^ occur at less than 3*ν*_esc_ (see Figure 17 in Appendix B for the full distribution of impact velocities) while planetesimal impacts occur at up to at most 20*ν*_esc_ in their simulations. Though their results apply strictly to embryos larger than 0.093 *M*_⊕_, the initial embryo mass in their simulations, we note that it is expected from theory that embryo–embryo collisions should take place at low velocities, a few times *ν*_esc_, due to dynamical friction with planetesimals (e.g., Goldreich et al. 2004). As we can see, this implies that disruptive collisions between embryos should be very rare as even in the more disruptive case of a head-on impact with an impactor-to-target mass ratio of 1 (left panel of Figure 1), the disruption criterion of Movshovitz et al. (2016; blue line) lies along the highest embryo–embryo collision velocities of Chambers (2013).

We emphasize that the blue disruption line shown in the left-hand panel of Figure 1 is the most favorable possible case. While this appears to show that it is possible to disrupt an Earth-mass body at 1 au, this would require a head-on impact with another Earth-mass object at an impact velocity equal to Earth’s orbital velocity (e.g., an eccentricity ≈ 1). This is an extremely unlikely impact configuration. Head-on impacts are strongly disfavored geometrically, as compared to grazing angles, and equal-mass collisions are also less likely than unequal-mass collisions. Similarly, an impact at close to the orbital velocity is very difficult to arrange as it requires an impactor that is plunging through the entire planetary system (Jackson et al. 2018). More typically, one would be considering disruption in terms of the green line in the right-hand panel—an unequal-mass collision at a glancing angle—and we can see that this lies far above the highest embryo–embryo collision velocities found by Chambers (2013). It is also notable that a large range of embryo–embryo collisions with *γ* = 0.1 occur at impact energies less than half the gravitational binding energy (hatched zone; Figure 1, right).

In contrast we can expect that planetesimal–planetesimal collisions will occur at absolute velocities similar to planetesimal–embryo collisions. The velocity dispersion in the planetesimals governs the collision velocities, and thus they occur at significantly higher values of *ν*_imp_/*ν*_esc_ because *ν*_esc_ for a planetesimal will be substantially smaller. Put in other terms, *ν*_esc_ is proportional to the mass of the colliding planetesimals (which are small), but *ν*_imp_ is proportional to the mass of the largest bodies in the dynamical “neighborhood” (which are comparatively much larger). As such, planetesimal–planetesimal collisions should generally be destructive and act to shut off planetesimal growth when the embryos are growing chaotically through giant impacts, as is generally assumed (e.g., Kenyon & Bromley 2016). However, the difference in size scale between asteroids and planetary embryos does not allow for them to be modeled explicitly in an *N*-body simulation of planet formation; instead, the full population of asteroids and small bodies are often modeled as fewer “super particles” that represent the much larger distribution.

In sum, giant-impact scaling laws must account for complexities, distinct from their classical cratering-physics counterparts, in both geometry and the energetics of the collision in order to make accurate predictions which are necessary and relevant for *N*-body planetary evolution codes. In the following section, we review the landscape of literature on giant-impact scaling laws, and the collision regimes for which they are appropriate, in order to provide the context for our contribution to the subject.

## 3. Existing Scaling Laws

Because we are studying giant impacts in the context of the final assembly of planets, it is clear that the mass of the largest body that emerges from the impact event, and perhaps the second, are important properties. A number of authors have developed scaling laws to predict remnant masses from pre-impact conditions. Housen & Holsapple (1990) provide a framework that most giant-impact scaling law literature is constructed upon, where the catastrophic disruption threshold for laboratory-scale to planetary-scale collisions is described in terms of a combination of the momentum and/or the energy of the collision. However, as discussed by Movshovitz et al. (2016; see their Appendix), the scaling laws are not appropriate for similar-sized collisions and, at least for the gravity regime, simple energy scaling is adequate. In particular, we highlight that the dimensional analysis approach used by Housen & Holsapple (1990) to develop their framework relies on a point-source approximation. This is a similar approximation in scaling relations for cratering, the development of which is well described by Holsapple (1993). Similar-sized collisions are, in stark contrast, not point-source interactions, and as such we should not expect that a framework derived in the point-source limit will continue to apply. Thus, scaling laws continue to evolve from early adaptations in the literature.

### 3.1. Benz & Asphaug (1999)

Benz & Asphaug (1999) focused on the catastrophic disruption threshold, in the context of collisions in the asteroid belt. They examined the transition from small-scale collisions (centimeters to meters in size), where the disruption outcome is governed by the material strength of the bodies, to large-scale collisions (kilometers in size), where the disruption outcome is dominated by self-gravity. To do so, they employed the SPH method with self-gravity and a model for the dynamical fracture of brittle material (Benz & Asphaug 1994) to simulate collisions into targets ranging from 1 cm to 100 km in diameter. They employed the Tillotson equation of state to model both basalt and water-ice targets.

Importantly, Benz & Asphaug (1999) discuss the distinction between what constitutes the “largest remnant” produced by strength-dominated collisions and gravity-dominated collisions. The largest remnant in strength-dominated collisions is the largest monolithic rock at the top of a size distribution of other fragments. In contrast, the largest remnant in gravity-dominated collisions is an accumulation of an array of gravitationally bound materials: intact monolithic fragments, fluidized debris, vapor-rich disk, etc.

Benz & Asphaug (1999) were focused primarily on the conditions of the asteroid belt, where the velocity dispersion of the swarm of small bodies can be well constrained, thus impact velocity was held constant. For a given target mass, they determined the disruption thresholds by holding both the impact angle and velocity constant while allowing the impactor size to vary, effectively probing different ranges of impact energy; because of this methodology, the impactor-to-target mass ratio (Equation (2)) required to disrupt the target was larger than 1 in some cases (Benz & Asphaug 1999). They limited their study to 3 and 5 km s^−1^ for basalt, and 0.5 and 3 km s^−1^ for ice.

They find that gravity-dominated bodies tend to become weaker as they decrease in size, with the weakest bodies being ~300 m in diameter, whereas bodies smaller than this become more resistant to disruption as the effects of material strength (tensile strength in their model) dominate. The scaling law they develop for both regimes is used widely and takes on the following form:

(4) $$Q_{\text{D}}^{*}=Q_{0}{\left(R_{\text{tar}}\right)}^{a}+B_{\rho }{\left(R_{\text{tar}}\right)}^{b},$$

where *Q*_0_, *a, B*, and *b* are fitted parameters. However, the fitted parameters are relevant for specific disruption velocities which were held constant, thus we caution the use of this scaling law outside of its intended context, for example, to solve for the disruption velocity given a constant *M*_tar_ and *M*_imp_ or to apply the relation to planet-scale collisions.

**Figure 2.**
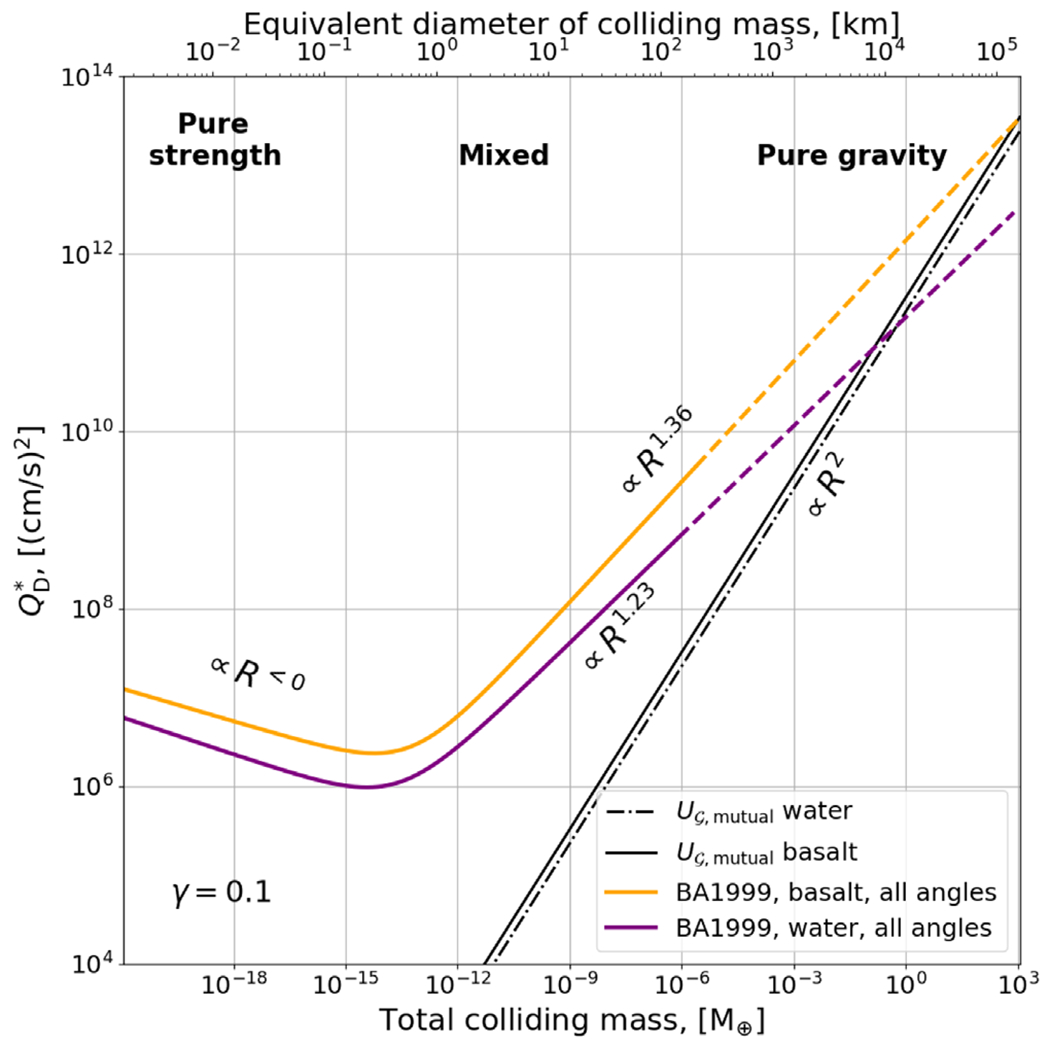
The catastrophic disruption threshold energy from Benz & Asphaug (1999) compared to the mutual gravitational binding energy for the colliding bodies with *γ* = 0.1. Dark red and orange lines are water-ice and basalt collisions that included a strength model and self-gravity, averaged over data for several angles and impact velocities. The black solid and dotted–dashed lines show the mutual gravitational binding energy for basalt (*ρ* = 3000 kg m^−3^) and water (*ρ* = 1000 kg m^−3^) bodies. Collisions with impact energy below the black lines cannot result in appreciable disruption, but the true disruption threshold likely lies above them.

In Figure 2, we compare the scaling law of Benz & Asphaug (1999) against the mutual binding energy of two colliding bodies. The slope of the gravity-regime term in Equation (4) (right-hand side) is shallower than that of the gravitational binding energy. This demonstrates that there exists a transitional regime, which we denote as “mixed,” where the collision outcome is dominated by both gravity and strength, and the slope must evolve from ~1.2–1.4 to 2 so as to not predict disruptive collisions with impact energies well below the gravitational binding energy. Because Benz & Asphaug (1999) include a strength model in their SPH simulations, they implicitly allow for the existence of such a mixed regime. In comparison, it is no surprise that scaling laws in the gravity-only domain, explicitly excluding material strength, (e.g., Movshovitz et al. 2016 and our work), report scaling laws proportional to *R*^2^. The slopes of the disruption thresholds in the mixed region have also been recently explored as a function of different strength models; the inclusion of dissipation by friction, for example, has shown that there are measurable differences in the catastrophic disruption threshold at sizes up to ~100 km (e.g., Jutzi 2015).

Benz & Asphaug (1999) also introduced a functional relationship for the mass of the largest remnant in a giant impact,

(5) $$\frac{M_{\mathrm{LR}}}{M_{\mathrm{tar}}}=-s\left(\frac{Q}{Q^{*}}-1\right)+0.5,$$

which was an important basis on which future scaling laws for remnant masses were based. Owing to the simplicity of this relation and Equation (4), they are used in numerous numerical studies of planet formation, and frequently well outside the parameter space that was studied.

Although our work is primarily focused on the collisions of gravity-dominated bodies, as is relevant to late-stage planet formation, understanding collisional outcomes in the transitional regime between strength- and gravity-dominated collisions is an area of important ongoing work. Collisions at the kilometer to tens of kilometers scale is critical in understanding the formation of small bodies, such as Vesta (e.g., Jutzi et al. 2013), irregularly shaped comets (e.g., Jutzi & Asphaug 2015), and asteroid collisional families (e.g., Jutzi et al. 2010, 2019). However, incorporating collisions of billions of small bodies and remnants in planet formation codes is still out of reach for even the most sophisticated *N*-body schemes.

### 3.2. Stewart & Leinhardt (2009)

Stewart & Leinhardt (2009) developed scaling laws to predict the mass of the largest remnant and focused on the catastrophic disruption threshold. They simulated low-velocity collisions between gravitationally bound granular aggregates (rubble piles) using the *N*-body code PKDGrav (e.g., Leinhardt et al. 2000; Richardson et al. 2000; Stadel 2001). The particles are indestructible and undeformable, and the contact physics were governed by restitution. Thus, the bodies are gravity dominated with no intergranular cohesion, no calculation of impact shock physics, and no measurable or evolvable thermodynamic states.

Stewart & Leinhardt (2009) studied aggregates with diameters *D* = 2, 20, 100 km undergoing head-on collisions at subsonic speed velocities (1–300 m s^−1^) and used two impactor-to-target mass ratios, *γ* = 0.03 and *γ* = 1. Disruption threshold data from several other studies were also used to fit scaling laws in Stewart & Leinhardt (2009), including simulations of strength-dominated collisions and laboratory studies of the disruption of approximately centimeter-sized targets.

To account for scenarios that involve collisions of objects with disparate densities, they developed a “normalized radius,”

(6) $$R_{\text{C}1}={\left(\frac{3}{4\pi }\frac{M_{\text{tot}}}{1\;\text{g}\;{\mathrm{cm}}^{-3}}\right)}^{1/3},$$

which represents an uncompressed sphere of water with mass *M*_tot_ = *M*_tar_ + *M*_imp_. Because giant impacts involve impactors comparable in size to the target, which is not the case in classical cratering, the reduced mass kinetic energy (scaled by the total mass) was introduced,

(7) $$Q_{\text{R}}=\frac{1}{2}\frac{\mu \nu_{\text{imp}}^{2}}{M_{\text{tot}}},$$

where *μ* is the reduced mass,

(8) $$\mu =\frac{M_{\text{tar}}M_{\text{imp}}}{M_{\text{tot}}},$$

and *ν*_imp_ is the impact velocity. Like Equation (3), the units of *Q*_R_ are in specific energy, but using a different normalization, *Q*_R_ ∝ *γ*/(1 + *γ*)^2^, whereas *Q* ∝ *γ*.

Under their scheme, the disruption threshold energy, $Q_{\mathrm{RD}}^{*}$, for a given set of colliding bodies is $Q_{\mathrm{RD}}^{*}=Q_{\text{R}}$ when $M_{\text{LR}}=\frac{1}{2}M_{\text{tar}}$. Based on the framework of Housen & Holsapple (1990), who developed disruption criteria for bodies in the strength and gravity-dominated regime, Stewart & Leinhardt (2009) reported a velocity-dependent relationship for $Q_{\mathrm{RD}}^{*}$,

(9) $$Q_{\mathrm{RD}}^{*}=q_{\text{s}}R_{\text{C}1}^{9\bar{\mu }/\left(3-2\phi \right)}\nu_{\text{imp}}^{\left(2-3\bar{\mu }\right)}+q_{g}R_{\text{C}1}^{3\bar{\mu }}\nu_{\text{imp}}^{2-3\bar{\mu }},$$

where in this case $\bar{\mu }$ is a fitted material parameter between $1/3⩽\bar{\mu }⩽2/3$ (with $\bar{\mu }=1/3$ representing pure “momentum scaling” and $\bar{\mu }=2/3$ representing pure “energy scaling”) and *ϕ* is a flaw distribution parameter that ranges from 6 to 9 depending on the material. The first term of Equation (9) has a negative slope, as appropriate for the strength regime (see Figure 2) and the second has a positive slope, as appropriate for the gravity regime. They find that $\bar{\mu }=0.4$, *q*_s_ = 500, and *q_g_* = 10^−4^ provides a good fit to simulations of gravity-dominated collisions (these values require *ν*_imp_ and *R*_C1_ to be in cgs units). Different constants were fit for laboratory experiments in the strength regime: *q*_s_ = 7 × 10^4^, *q_g_* = 10^−4^, $\bar{\mu }=0.5$, and *ϕ* = 8. Stewart & Leinhardt (2009) also report their catastrophic disruption threshold $Q_{\mathrm{RD}}^{*}$ (Equation (9)) for the gravity regime assuming pure-energy scaling ($\bar{\mu }=2/3$),

(10) $$Q_{\mathrm{RD}}^{*}=aR_{\text{C}1}^{2},$$

where *a* = (1.7 ± 0.3) × 10^−6^ and (5.3 ± 1.8) × 10^−6^ for equal-mass projectiles and small projectiles respectively; the velocity-dependent term drops out in this case and the relation is proportional to $R_{\text{C}1}^{2}$, as appropriate for the gravity regime (see black lines in Figure 2). Moreover, Stewart & Leinhardt (2009) provided a scaling law for the mass of the largest remnant *M*_LR_, similar to Equation (5) developed in Benz & Asphaug (1999),

(11) $$M_{\text{LR}}/M_{\text{tot}}=1-0.5Q_{\text{R}}/Q_{\mathrm{RD}}^{*},$$

that holds well for impact energies with $Q_{\text{R}}/Q_{\mathrm{RD}}^{*}<2$.

Although Stewart & Leinhardt (2009) demonstrate that this disruption criteria is robust for low-velocity collisions of self-gravitating aggregates, Equation (9) does not include dependence on impact angle. To that end, they note that $Q_{\mathrm{RD}}^{*}$ values seem to decrease by ~10% when *θ*_imp_ = 45°.

### 3.3. Leinhardt & Stewart (2012)

Leinhardt & Stewart (2012) developed scaling laws with parameters that were fit independently for small bodies (with and without strength) and large, hydrodynamic (strengthless) bodies, some of which included differentiated bodies (Leinhardt & Stewart 2012, Figure 12 and Table 3 therein). Their scaling law fit to small bodies included new simulations of subsonic collisions that used target bodies 10 km in radius with four different impactor masses, all with a bulk density of 1 g cm^−3^. They expanded on the work of Stewart & Leinhardt (2009) by simulating impacts at four different impact angles, $\theta_{\mathrm{imp}}=0^{{}^{\circ}},22_{.}^{{}^{\circ}}8,49_{.}^{{}^{\circ}}4,71_{.}^{{}^{\circ}}3$.

Leinhardt & Stewart (2012) used the concept of “interacting mass” to resolve the fact that in grazing collisions some of the impactor (if it is large enough) may interact only minimally with the target body. The interacting mass is constructed to represent only the portion of the impactor that directly intersects the target (see Figure 2 of Leinhardt & Stewart 2012). It is important to note, however, that the kinetic energy of the interacting mass is reported differently between Leinhardt & Stewart (2012, Equation (12)) and the full derivation reported in Movshovitz et al. (2016, Equation (20)). We however do not examine the origin of this discrepancy, because, as will be described later, we choose to eschew the concept of the interacting mass altogether.

The catastrophic disruption threshold in Leinhardt & Stewart (2012) includes a dependence on the impactor-to-target mass ratio, *γ*, and $\bar{\mu }$,

(12) $$Q_{\mathrm{RD}}^{*}=Q_{\mathrm{RD},\gamma =1}^{*}{\left(\frac{1}{4}\frac{{\left(\gamma +1\right)}^{2}}{\gamma }\right)}^{\left(\left(2/3\bar{\mu }\right)-1\right)},$$

where $Q_{\mathrm{RD},\gamma =1}^{*}$ is the catastrophic disruption threshold for a head-on collision between equal-mass bodies. However, Movshovitz et al. (2016) demonstrated that this correction produces inaccuracies for small values of *γ*. In the case of off-axis collisions, an additional correction for the interacting mass is applied (Leinhardt & Stewart 2012, Equation (15)). The catastrophic disruption threshold term for equal-mass bodies takes on a form similar to that of Equation (10),

(13) $$Q_{\mathrm{RD},\gamma =1}^{*}=c^{*}\frac{4}{5}\pi \rho_{1}GR_{\text{C}1}^{2},$$

where c* is a fitted parameter and *ρ*_1_ = 1 g cm^−3^. Using these relations, in conjunction with Equation (11), Leinhardt & Stewart (2012) reported that the mass of the largest remnant can be predicted for a variety of types of bodies (e.g., hydrodynamic or granular) with adjustments to the fit parameters. For hydrodynamic bodies, c* = 1.9 ± 0.3, and for small bodies, c* = 5 ± 2 (in cgs units), a difference of about a factor of 2 with some overlap.

The mass of the second largest remnant is computed two different ways, under Leinhardt & Stewart (2012), depending on the scenario. In a relatively head-on scenario, the second largest remnant is merely the largest body in the cascade of impact debris, i.e., the second largest remnant is the top of the debris size distribution often modeled with a power law. In a hit-and-run scenario, the second largest remnant is the eroded impactor that continues downrange along with debris that can be described by a size distribution. At the top of the debris size distribution in this case lies the third largest remnant. To determine the mass of the second largest remnant in a hit-and-run collision per Leinhardt & Stewart (2012), the catastrophic disruption of criteria of the “reverse” impact is computed. Note that in this case the interacting mass for the reverse collision is intended to exclude the mass of the target that does not directly intersect the impactor. The scaling law of Equation (11) is then used to determine the mass of the second largest remnant for any impact energy. Additional treatment for “supercatastrophic” collisions and other functionalities are also provided therein.

### 3.4. Movshovitz et al. (2016)

Movshovitz et al. (2016) paid particular attention to the catastrophic disruption threshold and determined the appropriate scaling law variables for the gravity-dominated regime to predict disruption. They simulate collisions at high energy, which allows for a direct interpolation of the catastrophic disruption threshold energy, providing an overall more accurate prediction than extrapolative methods. They find that the reduced mass impact energy, *K* = *Q*_R_*M*_tot_, is an ideal variable for scaling catastrophic disruption in the gravity regime (pure-energy scaling). Their disruption threshold K* is a multiple of the gravitational binding energy of the two-body system at the point of collision, $U_{G,\mathrm{mutual}}$; we introduce $U_{G,\mathrm{mutual}}$ formally later. Moreover, as stated in the previous section, Movshovitz et al. (2016) used a correction factor which removes the noninteracting mass of the collision from the computation of *K*; once the correction factor is applied, the kinetic energy is denoted as *K_α_*, and the catastrophic disruption data under this definition more tightly follow a power law. However, even when using this correction factor, they still found that the prefactor on K* is a function of *θ*_imp_, so additional empirical functionality is needed. Thus, we utilize the scaling parameters of Movshovitz et al. (2016) and replace the interaction mass correction by an empirical relationship.

## 4. Methodology

To understand how the outcomes of giant impacts vary under a variety of pre-impact conditions, we simulated collisions of similar-sized bodies using the 3D SPH code SPHLATCH (Reufer 2011). This code was designed specifically for handling giant impacts using a Barnes–Hut tree-based self-gravity calculation and can use the aneos, m-aneos, and Tillotson equations of state. sphlatch has been well tested against standard test problems, such as the blast tube test (Sod 1978), and used in previous planet formation studies (Reufer et al. 2012; Asphaug & Reufer 2013, 2014; Emsenhuber et al. 2018), producing similar outcomes to other SPH codes when applied to standard scenarios such as Moon formation. Because we are focused on predicting the mass of remnants in the gravity regime of giant impacts, we assume a fluid rheology and self-gravity in our SPH simulations; however, sphlatch has recently been updated to include material strength as well (Emsenhuber et al. 2018). We use the common form of artificial viscosity (Monaghan 1992) with no artificial viscosity “switches” (e.g., Balsara 1995); these switches are used to combat erroneous activation of artificial viscosity, but can introduce other nonphysical effects (see Raskin & Owen 2016 for the effects of these switches on a test problem most relevant to planetary scenarios). We also use the standard SPH formulation, which is based on the differentiability of density. We recognize the fact that in the case of the post-impact disk in the Moon-forming collision, density-independent formulations (which rely on the differentiability of pressure) provide different results from standard SPH and can better resolve static features of planetary problems such as the density discontinuity at the core-mantle boundary (Hosono et al. 2016). However, additional corrections must be employed to accurately resolve shocks in density-independent schemes (Saitoh & Makino 2013), and the effect of this formulation on the masses of the largest remnants generated through collisions with strong shocks is unclear at this time. Nevertheless, it is important to point out that the outcome of SPH simulations of different numerical varieties can vary, sometimes significantly, for planetary problems. Hosono et al. (2019), for example, finds that their density-independent SPH formulation shows a significant discrepancy in the amount of Earth material incorporated in the post-impact disk in the Moon-forming collision; this result effectively upends the high level of misplaced confidence in planetary SPH simulations on this topic and is an important cautionary tale. Thus, we caution the overinterpretation of model results below the few to ~10% level and suggest additional caution where boundaries between materials or free space are concerned (e.g., post-impact disks). Thus, this work is focused primarily on the masses of remnants, where numerical sensitivities are minimal and numerical convergence can be extrapolated (Genda et al. 2015). We use a cubic spline kernel, and the smoothing length is adjusted to an optimum number of 50 neighbors. Sensitivity to the choice of artificial viscosity, SPH flavor, or spline on the mass of the largest remnants is outside of the scope of this work, and thus we find it appropriate to use the most common forms. The methodology for determining the mass of remnants and bound clumps from SPH simulation output is described in Emsenhuber & Asphaug (2019). Data for each simulated collision are also provided as supplementary material; in Appendix A, we provide a sample of the data tables and show grids of accretion efficiency (defined later) for the entire database.

### 4.1. Parameter Space

A number of variables influence the outcome of a giant impact. These parameters include the impact scale (total mass of the colliding bodies; *M*_tot_), mass ratio between the target and the impactor (*γ* = *M*_imp_/*M*_tar_), impact angle (*θ*_imp_). impact velocity (*ν*_imp_). material composition, thermal state, material strength, and pre-impact rotational states. As such, the complete parameter space is inherently very large, and it is beneficial to identify the range of conditions probable in the late stages of planet formation, while others are held constant.

“Composition” in itself hides a multitude of different avenues for variation. For example, two bodies may have a similar bulk geochemistry but differ in mineralogy due to differentiation or their thermal state. Colliding bodies that originated from different dynamical zones may have entirely different geochemical compositions as well. Moreover, few material equations of state exist for the range of mineralogy found in primitive and evolved planets, and even fewer SPH codes implement a large range of equations of state. In this study, we choose to examine bodies composed of three materials: quartz (an analog for mantle/crust silicates), iron (an analog for nickel–iron core material), and water. Quartz was chosen in particular because its equation of state is most up to date (Melosh 2007), and it has been used extensively in giant-impact studies (e.g., Canup & Asphaug 2001; Marcus et al. 2009; Asphaug & Reufer 2014). We simulate pairs of colliding planets with three material categories: homogeneous SiO_2_, two-layer SiO_2_–Fe, and three-layer H_2_O–SiO_2_–Fe; thermodynamic information is sourced from tabularization of the aneos and m-aneos equation of state (Reufer 2011). The SiO_2_–Fe component in both cases is in “chondritic” abundance, 70 wt% SiO_2_ and 30 wt% Fe. The three-layer planets are 50 wt% H_2_O. The water-rich planet composition is the same composition used by Asphaug & Reufer (2013) and dissimilar to that of Marcus et al. (2010) who used a 50 wt% H_2_O, 50 wt% serpentine composition; both used the aneos equation of state. The difference between the bodies used by these studies is a good example of the influence of assumptions regarding differentiation. The Marcus et al. (2010) bodies represent a partially differentiated state, whereas ours and those of Asphaug & Reufer (2013) are more analogous to a fully differentiated state.

Collisions between bodies with nonzero rotation, either inherited from the disk or from a previous off-axis impact (Agnor et al. 1999), are an inevitable phenomenon in late-stage planet formation. First demonstrated by Canup (2008) in simulations of the Moon-forming collision, pre-impact rotation of the colliding bodies can fundamentally change disruption and accretion dynamics. Properly examining pre-impact rotation requires the consideration of six independent parameters, the spin rates of the target and impactor, alongside four angles to describe the orientation of the spin axes relative to each other and the impact plane. As such, considering pre-impact rotation inherently comes with a large computational cost, requiring that other parameters are held constant (as was done in Canup 2008; Rufu et al. 2017). To allow the consideration of a larger number of other parameters, we thus choose to ignore pre-impact rotation and reserve this topic for future study.

**Figure 3.**
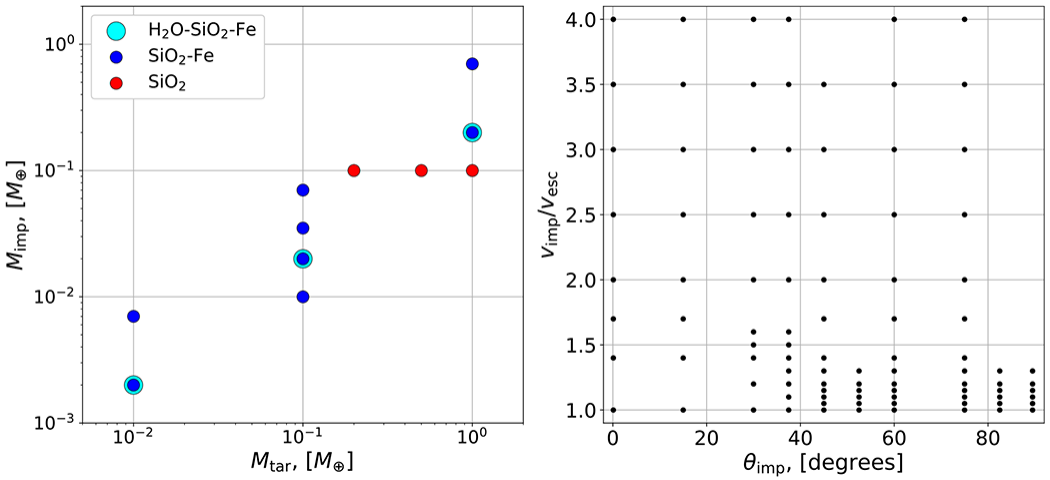
The parameter space of our giant-impact simulations in terms of the major input variables. The target mass (*M*_tar_) and impactor mass (*M*_imp_) combinations are shown in the left-hand panel. Red points indicate collisions between SiO_2_ bodies, blue points indicate collisions between SiO_2_–Fe bodies, and cyan points indicate collisions between H_2_O–SiO_2_–Fe bodies. At each point in the left-hand panel, simulations cover the full grid of impact angle (*θ*_imp_) and impact velocity normalized by the two-body escape velocity (Equation (1)) *ν*_imp_/*ν*_esc_ shown in the right-hand panel.

As discussed in Section 2, *N*-body simulations of late-stage formation of the terrestrial solar system demonstrate that planetary embryos collide at typically 1–2*ν*_esc_. Thus we conducted simulations across 1–4*ν*_esc_ to capture rare, higher impact velocity events, with finer resolution in the most probable 1–2*ν*_esc_ range. We simulated collisions between bodies with a range of impactor-to-target mass ratios from *γ* = 0.1 to 0.7, depending on the material. Our database spans several decades of *M*_tot_, from 10^−2^ to 1 *M*_⊕_, which spans escape velocities from a few to over 10 km s^−1^. We cover the entire range of possible impact angles from $\theta_{\mathrm{imp}}=0_{.}^{{}^{\circ}}1\;\mathrm{to}\;89_{.}^{{}^{\circ}}5$. Our simulations have a resolution of ~10^5^ nodes in the target body,^8^ as is widely used in giant-impact studies (e.g., Marcus et al. 2009, 2010; Canup 2011; Asphaug & Reufer 2013). Note that the 10^4^ particle *N*-body simulations performed by Leinhardt & Stewart (2012) are somewhat similar to 10^5^ node SPH simulations, due to the differences between the numerical schemes.^9^ The parameter space spanned by our simulations is depicted in Figure 3.

## 5. Our Scaling Laws

The aim of scaling laws is to reduce the input and outcome spaces, composed of thousands of node positions, velocities, thermal states, etc., to a few fundamental parameters, allowing for the broad outcome of the simulation to be predicted by simple functions of a small set of input and output parameters. Previous works have approached this from somewhat different perspectives, using different fundamental variables. Movshovitz et al. (2016) demonstrated the superiority of pure-energy seating for gravity-dominated collisions, and we find their structure to be intuitive, so we begin in a similar way. However, unlike Movshovitz et al. (2016), we aim to provide a set of predictive relationships for remnant and debris masses for use in *N*-body simulations and do not focus on constructing the most appropriate physical scaling law variables. To globally optimize our model, we employ an MCMC routine (Foreman-Mackey et al. 2013). The setup of the optimization scheme is reported in Appendix B. This scheme was chosen as it allows for a set of several equations and associated parameters to be globally optimized across multiple outputs in our database (largest remnant and runner mass in this case); however, other optimization schemes are also viable. The output of the scheme also allows for the assessment of degeneracy and the correlation of tit parameters, which provides important feedback in the development of empirical relationships.

The first parameter we use is the impact energy, which acts to disrupt the bodies in a collision,

(14) $$K=\frac{1}{2}\mu v_{\text{imp}}^{2},$$

where *μ* is described by Equation (8) and *ν*_imp_ is the impact velocity of the two bodies.

### 5.1. Gravitational Binding Energy

For collisions between large bodies, the total gravitational potential energy $U_{G}$ is the dominant contributor to the overall binding energy of the bodies (e.g., Benz & Asphaug 1999). The binding energy is thus described by

(15) $$U_{G}=-G\int_{0}^{R}M\left(r\right)m\left(r\right)\frac{dr}{r},$$

where *m*(*r*) is the mass of a shell of size *dr, M*(*r*) is the mass interior to the shell, and *R* is the radius of the planet. Assuming a constant density throughout the body, the equation simplifies to

(16) $$U_{G}=\frac{3}{5}\frac{GM^{2}}{R},$$

where *M* is the total mass of the body, and we use the convention that the binding energy is positive.

The binding energy of the system of impacting bodies must account for their separation at impact (e.g., Movshovitz et al. 2016),

(17) $$U_{G}=U_{G,\text{tar}}+U_{G,\text{imp}}+G\frac{M_{\text{tar}}M_{\text{imp}}}{R_{\text{tar}}+R_{\text{imp}}},$$

where the binding energy of the target and impactor, $U_{G,\mathrm{tar}}$ and $U_{G,\mathrm{imp}}$ respectively, can be computed either analytically (Equation (16)) or by integrating Equation (15), but the offset factor is an approximation.

#### 5.1.1. Compression and Density Stratification

The assumption of constant density yields a lower limit for the actual gravitational binding energy. As a planet increases in mass, the internal pressures rise and the materials begin to compress, resulting in density gradients even within layers of constant bulk composition. In the simulations of Movshovitz et al. (2016) and pkdgrav simulations of small bodies in Stewart & Leinhardt (2009) and Leinhardt & Stewart (2012), the bodies are small and compression is negligible. Here, however, we study bodies covering several orders of magnitude in mass, and even our smallest bodies are larger than the largest bodies used by Movshovitz et al. (2016). Combined with the three different compositions, two of which are layered, our simulations thus span a range of degrees of density stratification that may influence impact outcomes.

To quantify the degree of density stratification of the two bodies involved in the collision in a simple one-dimensional measure, we introduce the ratio of the analytical and numerical values of the gravitational binding energy of the two bodies,

(18) $$\Lambda =\frac{U_{G,a,\text{tar}}+U_{G,a,\text{imp}}}{U_{G,n,\text{tar}}+U_{G,n,\text{imp}}},$$

where the analytical value, $U_{G,a}$, is calculated using the constant-density approximation (Equation (16)). For individual bodies, the ratio of the analytic and numerical values, $U_{G,a}/U_{G,n}$, can reach ~85%. The bodies used in our study and their physical parameters are reported in Table 1. The value of Λ is smaller for more massive bodies as increased central pressures result in greater compression and transitions to high-pressure polymorphs. Note that even in the pure SiO_2_ bodies, compression under gravity and a solid-state phase transition results in a density gradient toward the center of the body. This produces minor discrepancies between $U_{G,\text{a}}$ and $U_{G,\text{n}}$.

**Figure 4.**
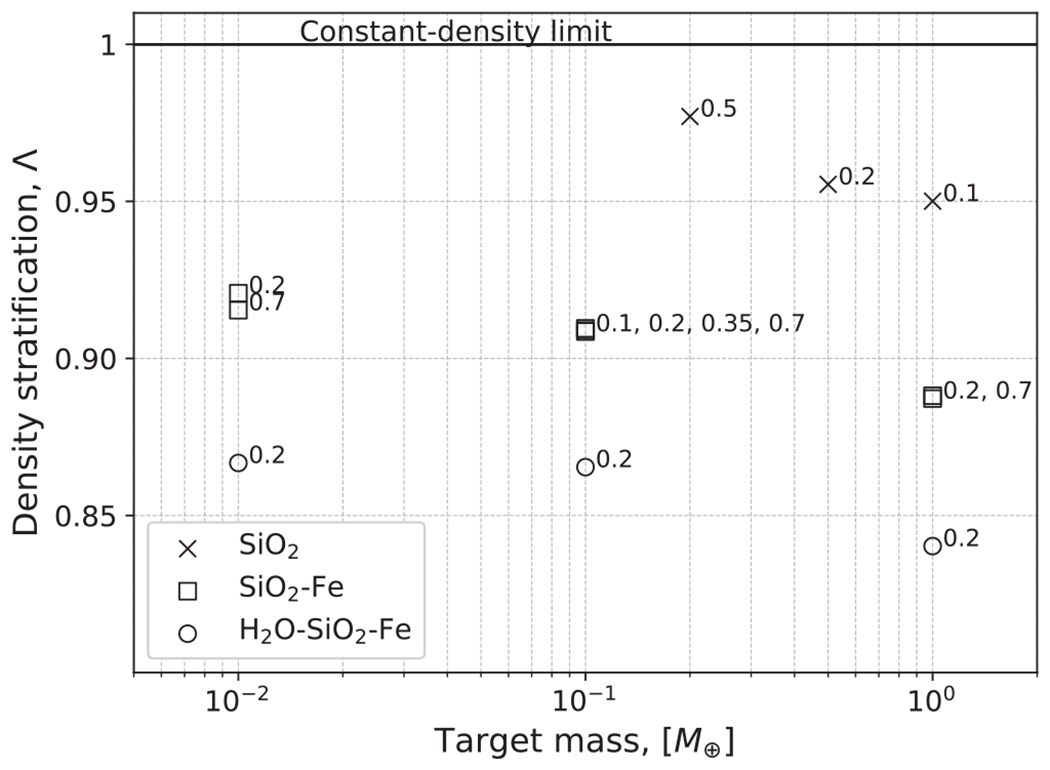
The ratio of the analytically and numerically determined binding energy of the target and impactor, Λ. This ratio represents the degree of density stratification of the two bodies involved in the collision, with Λ = 1 being the uncompressed, homogeneous density limit. The ×, square, and circle symbols represent the homogeneous SiO_2_, two-layer SiO_2_–Fe, and three-layer H_2_O–SiO_2_–Fe bodies, respectively. The impactor-to-target mass ratio is reported next to each point. The homogeneous silicate bodies approach the constant-density limit for small-enough masses; however, we note that these planets still include a solid-state phase transition and demonstrate minor levels of stratification.

We show the values of Λ for bodies in our study in Figure 4. Compression and density stratification cause deviations from the analytical binding energy, especially for the layered bodies, as expected. As we discuss in later sections, we find that this ratio is useful in helping predict hit-and-run collisions. We note, however, that there is degeneracy in this formulation. Bodies of different compositions may yield similar values of Λ, but so long as the onset of hit and run depends solely on mass distribution, the utility of this parameter is likely to hold reasonably well.

### 5.2. Predicting Hit and Run

Many studies (e.g., Agnor & Asphaug 2004; Asphaug et al. 2006; Kokubo & Genda 2010; Stewart & Leinhardt 2012) have shown that a substantial region of pre-impact conditions of similar-sized collisions result in hit and run. It is natural then that *N*-body studies have shown that such hit-and-run outcomes are common, accounting for around half of all giant impacts in many cases (e.g., Kokubo & Genda 2010; Chambers 2013). Delineating the transition between the merging/erosive and hit-and-run regime is thus clearly important.

Here we define the hit-and-run transition as the point in which the impactor continues downrange, largely unscathed. This transition presents itself as a step discontinuity, *ξ*_jump_, in the accretion efficiency parameter developed by Asphaug (2010),

(19) $$\xi =\frac{M_{\text{LR}}-M_{\text{tar}}}{M_{\text{imp}}}.$$

In a perfect merger, when the mass of the largest remnant *M*_LR_ is equal to the total mass of the colliding bodies, *M*_LR_ = *M*_tar_ + *M*_imp_ and *ξ* = 1. If the impact is a merger that produces escaping debris, with mass *M*_esc_ = *M*_imp_, then *M*_LR_ = *M*_tar_ and *ξ* = 0. Similarly, in a clean hit-and-run collision, where the impactor continues downrange unscathed, *M*_LR_ = *M*_tar_ and thus *ξ* = 0 as well. As such, taken on its own, accretion efficiency is degenerate with respect to hit-and-run and erosive collisions, i.e., the fate of the “runner’. Without knowledge of the second largest remnant mass, an erosive collision and a hit-and-run collision cannot be distinguished by *ξ* alone.

#### 5.2.1. The Impact Angle Threshold

**Figure 5.**
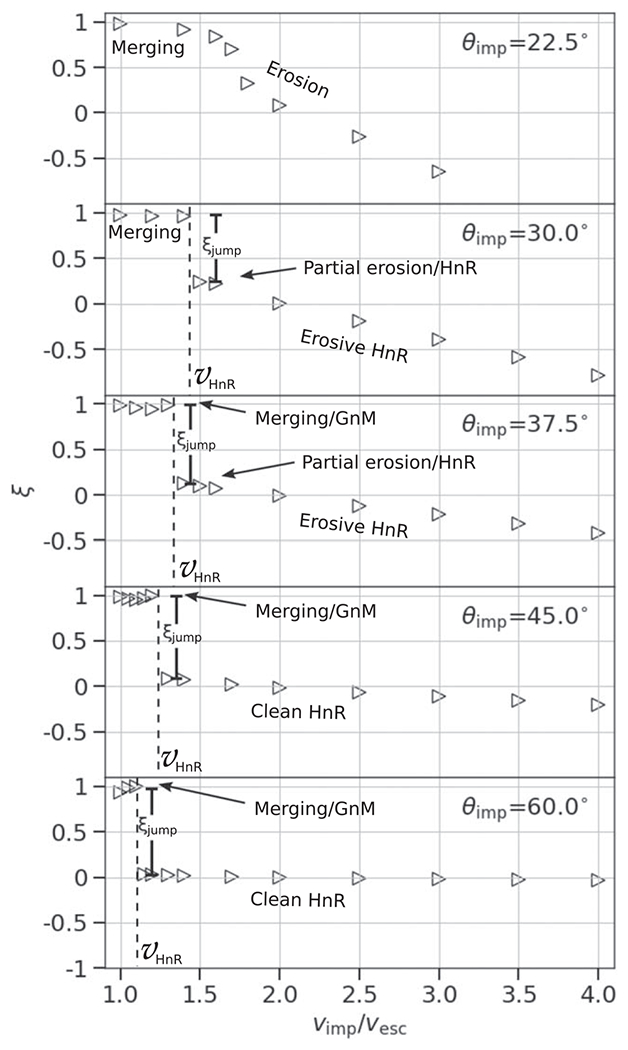
Accretion efficiency for simulations of rock-iron planets with *M*_tar_ = 0.2 *M*_⊕_ and *M*_imp_ = 0.1 *M*_⊕_ impacting at $\theta_{\mathrm{imp}}=22_{.}^{{}^{\circ}}5$, 30°. 45°, and 60°. The step discontinuity of *ξ* due to “runner disruption/hit and run” (*ξ*_jump_) is evident for $\theta_{\mathrm{imp}}>22_{.}^{{}^{\circ}}5$. For this combination of *M*_tar_, *γ*, and material type, the geometric threshold for hit and run has the bounds: $22_{.}^{{}^{\circ}}5<\theta_{\mathrm{HnR}}⩽30^{{}^{\circ}}$. The dashed line indicates the velocity threshold for hit and run. which is inversely related to *θ*_imp_.

At low impact angles and low velocities, the colliding bodies merge and produce minimal mass (a few percent of the total mass) to debris, so *ξ* ≈ 1. At low angles, the accretion efficiency also smoothly declines as a function of impact velocity, because more escaping debris is produced (see Figure 5, top panel). In contrast, at some threshold angle, a sharp, step-like discontinuity in *ξ* occurs (see second from top panel). This marks the point at which a large portion of the impactor mass does not accrete onto the target but is dispersed downrange, either as a debris field (“impactor disruption”) or as a relatively intact mass (hit and run). At larger angles, a majority of the parameter space between 1*ν*_esc_ and 4*ν*_esc_ is dominated by hit and run of a cleaner variety. However, as discussed in Section 5.2, a value of *ξ* ≈ 0 alone does not alone predict hit and run. For example, from Figure 5, we can see that at $\theta_{\mathrm{imp}}=22_{.}^{{}^{\circ}}5$ and *ν*_imp_ = 2*ν*_esc_, a value of *ξ* ≈ 0 is found, yet the hit-and-run discontinuity is not shown. In Appendix A, we show accretion efficiency across the entire database of SPH simulations and they demonstrate that the degeneracy exists for all combinations of *γ* and *M*_tar_; values of *ξ* ≈ 0 are present both in erosive (more head-on) collisions and hit-and-run (more glancing) collisions.

We show only one value of the impactor-to-target mass ratio in Figure 5, however, the hit-and-run angle, *θ*_HnR_ can vary depending on the impactor-to-target mass ratio. For similar-sized collisions, Asphaug (2010) defined the grazing angle, *θ_b_*, that represents the impact geometry at which the velocity vector drawn through the center of the impactor does not intersect the target,

(20) $$\theta_{b}=\sin^{-1}\;\left(\frac{R_{\text{tar}}}{R_{\text{imp}}+R_{\text{tar}}}\right).$$

For cases with *γ* ≪ 1, the impactor is colliding with what can be approximated as an infinite plane, whereas in a giant impact, the target is comparable in size to the projectile. This makes it common for the projectile core to miss the target core entirely in a typical giant impact, plowing instead through its less-dense mantle.

Due to the lack of a recipe for hit-and-run angle in the literature, several authors have implemented the grazing criterion (Equation (20)) to represent the angular threshold beyond which all collisions are considered hit and run (e.g., Chambers 2013; Quintana et al. 2016);^10^ that is, any collision that satisfies *θ*_imp_ > *θ*_b_, regardless of the impact velocities, is hit and run. However, Figure 5 demonstrates that the velocity at which hit and run occurs varies with impact angle. The grazing criterion simplification will overestimate hit-and-run collisions in the case of events that would actually be low-velocity graze-and-merge events; for example, the canonical Moon-forming giant impact would be hit and run according to that rule. Conversely, it will underestimate hit-and-run collisions in the case of high impact velocity; some collisions as steep as 15°–20°, close to head-on, can be hit and run under certain conditions (we demonstrate this later). Leinhardt & Stewart (2012) use Equation (20) in the construction of their scaling laws, alongside the velocity threshold criterion to account for the velocity-dependent behavior.

**Figure 6.**
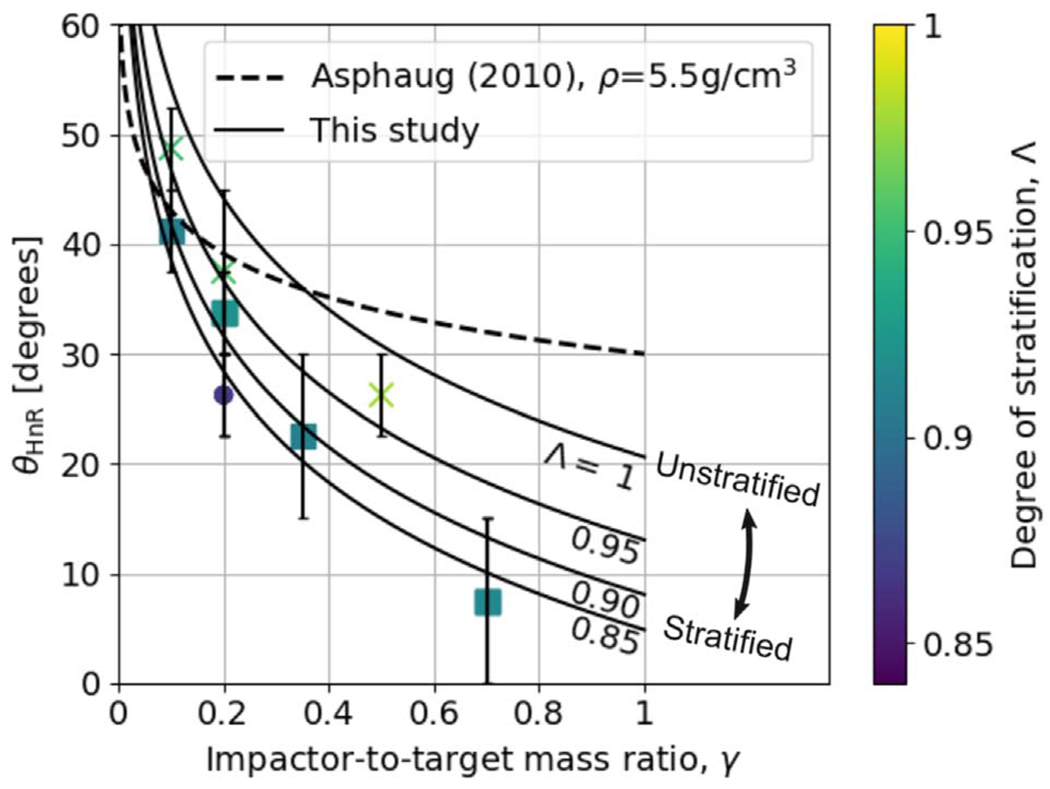
Angles at which collisions can transition from merging to hit and run as a function of impactor-to-target mass ratio, *γ*. The Asphaug (2010) grazing criterion is the dashed line (Equation (20)). The ×, □, and ○ symbols represent the homogeneous SiO_2_, two-layer SiO_2_–Fe, and three-layer H_2_O–SiO_2_–Fe bodies, respectively. Error bars extend from the angle at which hit and run does not occur (lower bound) to the angle at which it does (upper bound). Color represents the degree of density stratification. Planets with greater density stratification can hit and run at lower angles (closer to head-on geometry) as evidenced by the hit-and-run transition of the icy bodies (blue circle) for *γ* = 0.2, whereas uncompressed, homogeneous bodies require a more glancing geometry.

Our study includes a reasonably high-resolution sampling of *θ*_imp_, enabling us to directly estimate θ_HnR_ for the first time. For example, as shown in Figure 5, by having a fine resolution in *θ*_imp_ and *ν*_imp_, we are able to find that the angle at which hit and run may occur for this pair of bodies is between $22_{.}^{{}^{\circ}}5<\theta_{\mathrm{HnR}}⩽30^{{}^{\circ}}$, whereas Equation (20) would predict it to occur at $42_{.}^{{}^{\circ}}5$. However, determining the hit-and-run angle must take into account the mass of the second largest remnant (runner) because hit and run is described as when the impactor continues downrange somewhat unscathed, by our definition. We estimated the hit-and-run angle across the database of simulations, assuming hit and run is defined by (1) a discontinuity in *ξ* and (2) a relatively intact second largest remnant (*M*_2LR_ ⩾ 0.5*M*_imp_). Using these criteria, we show the relationship of *θ*_HnR_ with respect to pre-impact parameters in Figure 6. It is first immediately clear that the hit-and-run angle decreases as a function of *γ*, as predicted by Equation (20). This effect is recognized in the accretion efficiency data shown in Appendix A; the transition from merging to hit and run (*ξ* ≈ 1 to *ξ* ≈ 0) occurs at lower angles for larger *γ*.

In Figure 6, the hit-and-run thresholds appear to differ for the three material compositions, or equivalently, density stratifications. This is most noticeable at *γ* = 0.2, where there are data for all three compositions. Homogeneous bodies transition to hit and run at systematically larger angles than the rock-iron two-layer bodies, which hit and run at larger angles than the water-rich three-layer bodies. The merging regime (*ξ* ≈ 1) is larger, encompassing higher velocity collisions, in the less stratified bodies. Less stratified bodies also tend to undergo disruption of the impactor into a string of remnants that are subsequently accumulated gravitationally downrange. For example, simulations at *γ* = 0.2 between pure SiO_2_ bodies show *M*_2LR_ ≈ 0.5 *M*_imp_ at $\theta_{\mathrm{imp}}=37_{.}^{{}^{\circ}}5$ for a slim set of impact velocities; however, the runner is largely gravitationally accumulated debris. We can understand the origin of this trend physically as a more centrally condensed impactor can more easily suffer a collision and partial stripping of some of the outer layers while still retaining the bulk of its mass as a bound entity.

To account for the dependence of the hit-and-run angle on density stratification, we introduce the parameter Λ, defined in Section 5.1, in our empirical prediction of *θ*_HnR_:

(21) $$\theta_{\text{HnR}}=a\;\log_{10}\left(\gamma \right)+b\Lambda^{c},$$

where *a* = −33.8, *b* = 20.6, *c* = 8.9, and *θ*_HnR_ is in degrees. We note that the data in Figure 6 are illustrative; our model was optimized to *M*_LR_/*M*_tot_ and *M*_2LR_/*M*_imp_ via a weighted MCMC scheme, thus the scheme is agnostic to whether or not the impactor remained “relatively intact” throughout the collision or was gravitationally reaccumulated downrange. The optimization finds dependence on stratification as demonstrated by the values of *b* and *c* (see Appendix B and Table 2 for a list of all fitted parameters). It is also immediately apparent that hit and run generally occurs at lower angles than predicted by models that assume *θ*_HnR_ = *θ_b_*. particularly for larger values of *γ* and for stratified planets.

We note that Equation (21) can only be accurate to ±15°, because this is the angular resolution of our database and is close to the variation between the material types. Moreover, the transition to hit and run is not binary; instead, collisions transition from “impactor disruption” to hit and run semi-smoothly, depending on density stratification; this transition is also on the order of ~15°. Future work involving a larger number of different density stratifications, particularly at large values of *γ*, would be needed to validate and better characterize the functionality of this effect. We acknowledge the possibility that the effect may be due, at least in part, to thermodynamic effects; the different density stratifications in this study are produced by combinations of different materials, with different equations of state. For example, the most stratified planets are also the most volatile (water) rich, providing a different thermodynamic circumstance in the collision than the pure SiO_2_ or SiO_2_–Fe bodies.

#### 5.2.2. The Impact Velocity Threshold

**Figure 7.**
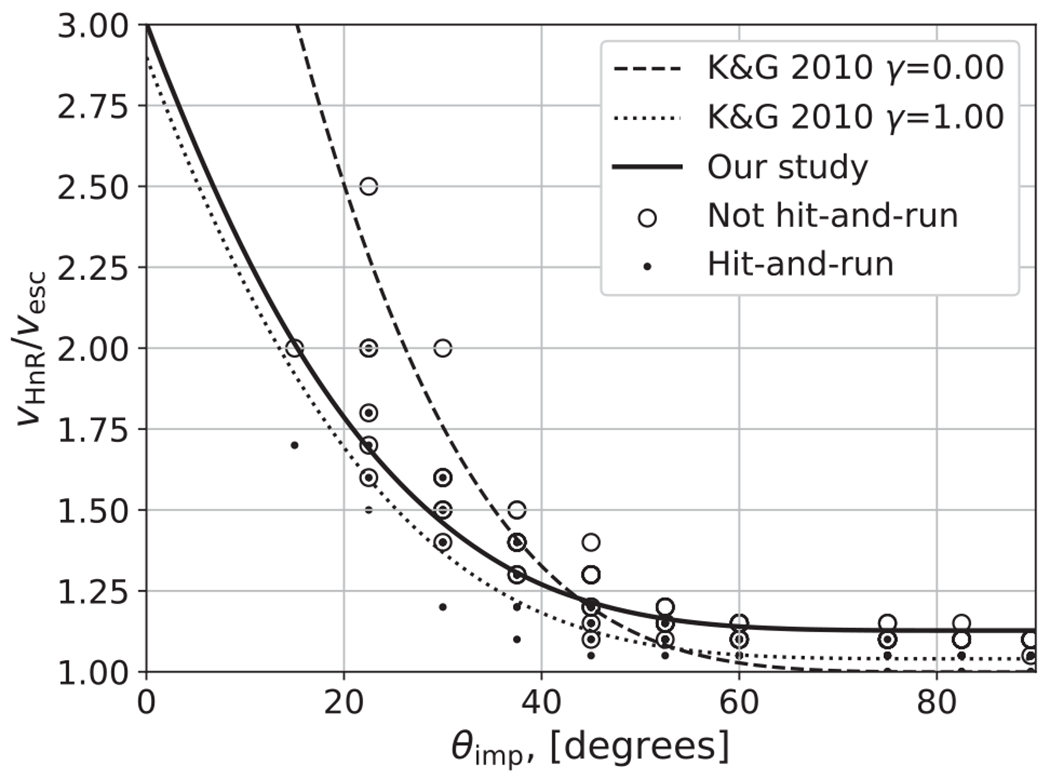
The relationship between *θ*_imp_ and *ν*_HnR_. Closed circles represent the highest impact velocity at which hit-and-run does not occur and open circles represent the lowest impact velocity at which hit-and-run occurs for each combination of *θ*_imp_, material type, *γ*, and *M*_tar_. The dashed curves are the velocity criteria of Kokubo & Genda (2010) (Equation (22)) for the end-member scenarios of *γ* = 0 and 1.0. The solid curve is our *γ*-independent relation (Equation (23)).

The criterion of *θ*_imp_ > *θ*_HnR_ is a necessary, but not sufficient, condition to define the transition to hit and run. For example, in the free-fall velocity limit (*ν*_imp_ = *ν*_esc,mutual_), the impactor is bound and is thus guaranteed to merge with the target regardless of impact angle. The dashed lines in Figure 5 demonstrate that the hit-and-run velocity threshold decreases at glancing geometries, due to less of the impactor being involved in the collisional interaction. Kokubo & Genda (2010) fit a hit-and-run criterion (their Equation (16)) as a function of both *θ*_imp_ and *γ*,

(22) $$\frac{v_{\text{HnR}}}{v_{\text{esc}}}=2.43\Gamma {ϴ}^{5/2}-0.0408\Gamma +1.86{ϴ}^{5/2}+1.04,$$

where Γ = (1 − *γ*)/(1 + *γ*) = (*M*_tar_ − *M*_imp_)/*M*_tar_ and ϴ = 1 − sin(*θ*_imp_). We found limited evidence for dependence of the impact velocity threshold on the impactor-to-target mass ratio (*γ*), so we use the *γ*-free formulation

(23) $$\frac{\nu_{\text{HnR}}}{\nu_{\text{esc}}}=d{\left(1-\sin\left(\theta_{\text{imp}}\right)\right)}^{5/2}+e,$$

where *d* = 1.88 and *e* = 1.13, and *e* represents the hit-and-run velocity in the limit of *θ*_imp_ → 90°. As shown in Figure 7, Equation (22) and (23) resolve the hit-and-run velocity threshold with similar accuracy. However, using Equation (22) without a geometric threshold would allow for near-head-on hit-and-run collisions if impact velocity is high; these conditions are more likely to lead to the disruption of the impactor than hit and run. For similar reasons described in Appendix C, we rule out the angular momentum criterion of Jutzi & Asphaug (2015) as a standalone predictor of hit and run across all angles and velocities. We also examined the extent to which the velocity transition to hit and run may be dependent on impact scale, i.e., whether Equation (23) requires dependence on *M*_tot_. To do so, we leverage the fact that the H_2_O–SiO_2_–Fe simulations span three orders of magnitude in total mass and use a constant *γ*. We find that the onset of hit and run is potentially variable at the ±0.05–0.1*ν*_esc_ level (just at the resolution of our database) and is thus minimal. We also find that the velocity criterion is similarly insensitive to Λ, which was checked by examining H_2_O–SiO_2_–Fe and SiO_2_–Fe simulations at *γ* = 0.2.

### 5.3. On the Nuances of Graze and Merge

Graze and merge is a phenomenon observed in the study of the giant-impact formation of the Moon (e.g., Cameron 2001) and in some models of the Haumea system (e.g., Leinhardt et al. 2010). In a giant impact although the impactor may have sufficient energy to escape the two-body system, the relatively minor collisional interaction in a grazing collision can reduce the kinetic energy sufficiently such that the impactor becomes bound to the target. The net effect is that graze-and-merge collisions involve two separate collisions, one with *ν*_imp_/*ν*_esc_ ⪆ 1 and another, occurring soon after or days later, with *ν*_imp_/*ν*_esc_ < 1 (Agnor & Asphaug 2004).

Physically, graze and merge itself manifests in several ways. The two bodies can remain relatively near one another, in a series of tidal–collisional interactions or the impactor can continue downrange as a temporary runner, only to reaccrete some time later. As graze-and-merge collisions exist on a slim phase boundary whose outcome is critically dependent on the brief, minor interactions between small amounts of mass (and thus few SPH nodes), they are an outcome regime that is highly sensitive to numerical aspects (rounding error, time-stepping schemes, SPH smoothing kernels, etc.) and initial conditions (density stratification, initial setup of the orbits, equation of state, etc.). Indeed, Genda et al. (2012) found that different initial thermal states can change the transition between merging and hit and run. Due to the more drawn-out process of a graze-and-merge collision, it is also important that the simulation is allowed to run for long enough or the outcome may not be converged. We do not attempt to provide empirical laws regarding graze-and-merge collisions as empirical relationships for graze and merge are likely to be an outcome very discrepant from one SPH implementation to another; this was similarly avoided in Leinhardt & Stewart (2012). However, because the graze-and-merge regime occurs at impact angles and velocities common in embryo–embryo collisions, it is an important area of study that requires detailed comparisons of SPH formulations and consideration for initial conditions.

### 5.4. Hit-and-run Efficiency

In a “clean” hit and run, the target and impactor are both minimally disrupted, i.e., *M*_LR_ ≈ *M*_tar_ and *M*_2LR_ ≈ *M*_imp_, where *M*_run_ is the mass of the second largest remnant in hit and run. From Equation (19), it follows that *ξ* = 0 in this case. However, as shown in the 30° case in Figure 5 and in Asphaug (2010, Figure 8), the target can be partially eroded in a hit-and-run collision, so *ξ* > 0 is achievable. This is labeled “Partial erosion/HnR”; however, for a slim range of geometries near *θ*_imp_ ≈ *θ*_HnR_, the impactor may be disrupted, potentially to be reaccumulated downrange. As the impact angle increases, the hit-and-run outcome becomes more “clean” and the target retains a greater amount of its pre-impact mass. Simultaneously, in terms of the runner, as impact angle increases, it is disrupted to a lesser extent.

**Figure 8.**
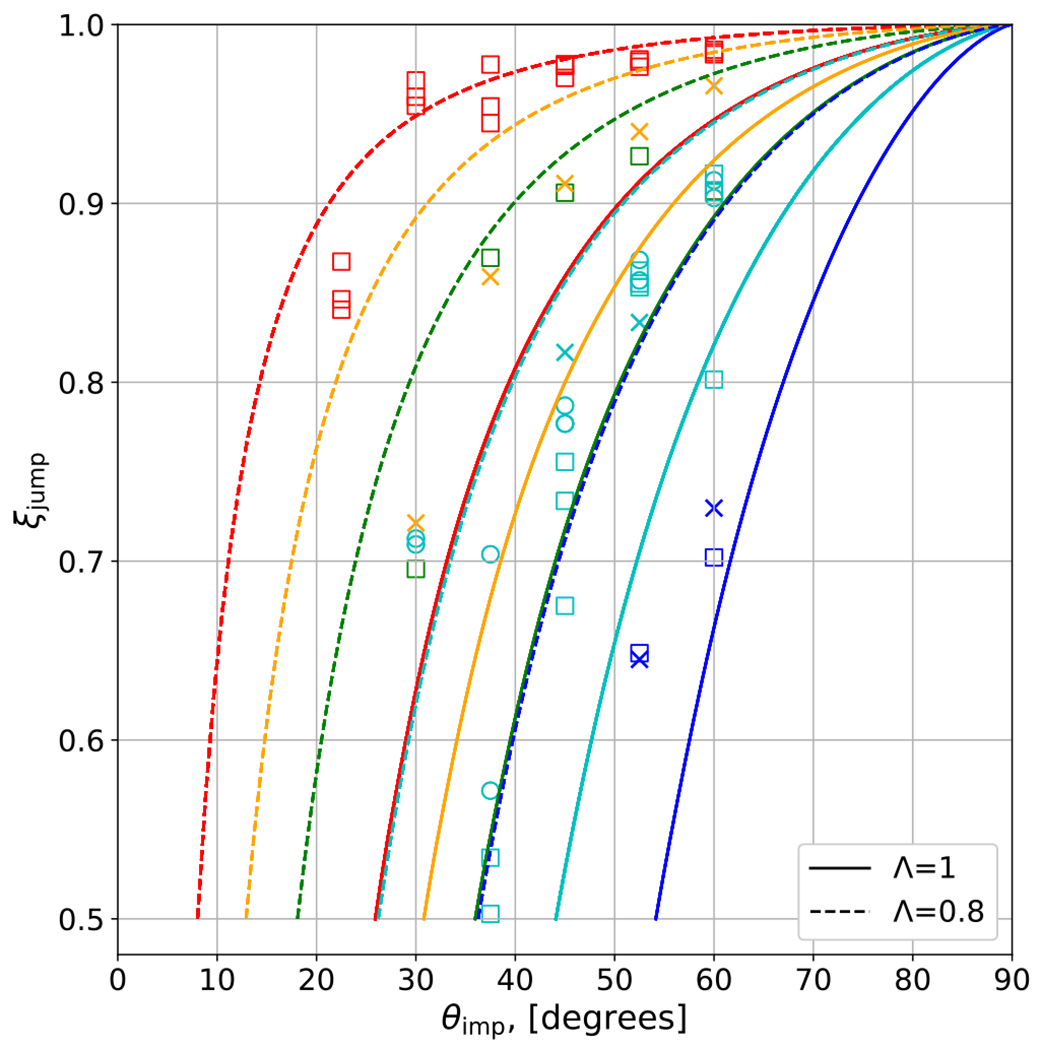
The jumps in accretion efficiency that occur in the transition from merge/graze and merge to hit and run. Red, orange, green, cyan, and blue symbols represent jumps determined by hand for our *γ* = 0.7, 0.5, 0.35, 0.2, and 0.1 simulations. The ×, □, and ○ symbols represent the homogeneous SiO_2_, two-layer SiO_2_–Fe, and three-layer H_2_O–SiO_2_–Fe bodies, respectively. Values were computed “by hand” from the data, and high angle data were excluded as it suffers from stochastic effects from graze and merge. Plotted data are shown for illustration purposes; the lines indicate the MCMC optimization of Equation (24) to data of *M*_LR_ and *M*_2LR_ across the entire database of SPH simulations.

To account for the behavior of the efficiency of hit and run, we fit an empirical model that predicts the discontinuity in accretion efficiency, *ξ*_jump_, seen by the example in Figure 5. As shown in Figure 8, we find that the jump in accretion efficiency between merge/graze and merge and hit and run is described with reasonable accuracy by the following function:

(24) $$\xi_{\text{imp}}=1-0.5{\left(\frac{\theta_{\text{HnR}}\left(90^{\circ }-\theta_{\text{imp}}\right)}{\theta_{\text{imp}}\left(90^{\circ }-\theta_{\text{HnR}}\right)}\right)}^{f},$$

where *f* = 1.42, *θ*_HnR_ is described by Equation (21), and angles are in degrees. We find that collisions of large impactor-to-target mass ratio tend to have “cleaner,” more efficient hit-and-run transitions, i.e., *ξ*_jump_ ≈ 1 for a large range of angles. This is likely due to two reasons: (1) for a fixed impact geometry, smaller impactors have a greater fraction of their own mass interacting in the collision and (2) the discrepancy between the gravitational binding energies is greatest for small-*γ* scenarios, so the impactor is less robust against the strong tidal interaction. Accretion efficiency data shown in Figure A illustrates this effect where the hit-and-run transition is increasingly diffuse for disparately sized bodies (small-*γ* collisions).

To obtain a fit that produces a smooth transition in *ξ*, a feature that would be provided by purely data-driven methods, the optimization would need to be performed with the prefactor (currently 0.5) as a free parameter. However, our model is designed to differentiate the occurrence of a runner from simply the largest remnant in a debris cascade, which is an important distinction or “switch” for *N*-body implementations, as the runner and the debris held are dynamically, morphologically, and thermodynamically distinct objects.

### 5.5. Maximum Mass of Remnants

In the non-hit-and-run regime, the maximum achievable mass of the largest remnant is $M_{\text{LR}}^{\prime }=M_{\text{tot}}$, although we note that all simulations, even those near *ν*_imp_/*ν*_esc_ ~ 1, involve some amount of escaping debris. In the hit-and-run regime, the maximum achievable mass of the largest remnant is $M_{\text{LR}}^{\prime }=M_{\text{tot}}-\xi_{\text{jump}}M_{\text{imp}}$, where *ξ*_jump_ is provided by Equation (24). It follows that $M_{\text{LR}}^{\prime }$ is a piecewise function, dependent on whether or not the collision is a hit and run:

(25) $$M_{\text{LR}}^{\prime }=\{\begin{array}{ll} M_{\text{tot}} & \text{not HnR}\\ M_{\text{tot}}-\xi_{\text{imp}}M_{\text{imp}} & \text{HnR}. \end{array}$$

We find that the following scaling law is sufficient for predicting the mass of the largest remnant:

(26) $$M_{\text{LR}}=M_{\text{LR}}^{\prime }\left(1-\frac{K}{\alpha U_{G}}\right),$$

where *K* is computed using Equation (14) and *α* is a fitted parameter. The largest remnant will be half of its maximum mass once $K=K^{*}=\alpha U_{G}/2$. The value of *M*_LR_/*M*_tot_ should be zero where Equation (26) predicts negative values, though as discussed in Section 2.1, highly disruptive scenarios are not common in late-stage planet formation.

**Figure 9.**
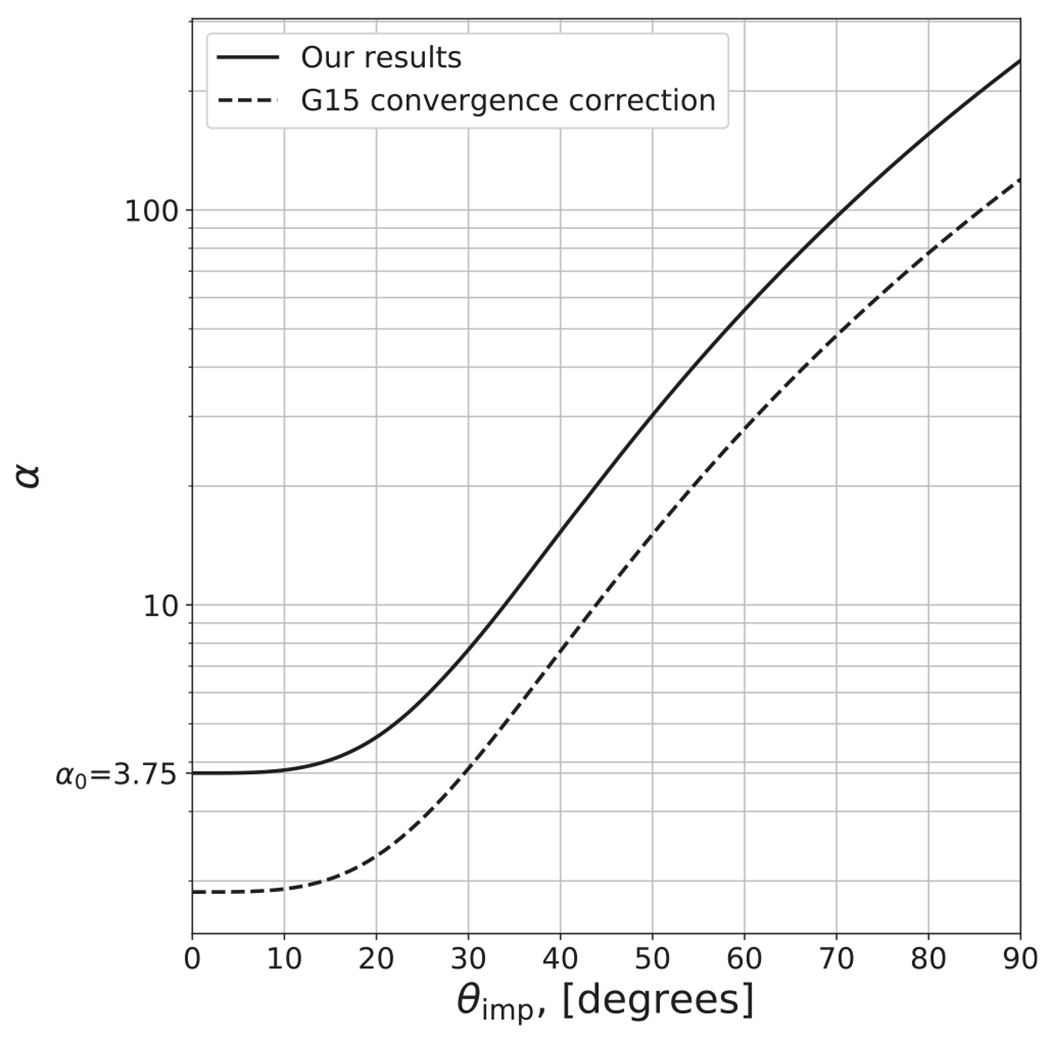
The behavior of *α* (Equation (27)) as a function of impact angle. We show a 50% correction due to the unconverged nature of disruption thresholds in SPH simulations, labeled “G15” (Genda et al. 2015).

We found the behavior of *α* is best described as an exponential function of *θ*_imp_,

(27) $$\alpha =g\theta_{\text{imp}}^{h}+\alpha_{0},$$

where *g* = 10^−4,90^, *h* = 3.72, and *α*_0_ = 3.75. We point out that *α* has very large values at glancing angles (see Figure 9); for example, *α* differs by roughly a factor of 5 for collisions between *θ*_imp_ = 0° and 45°. Thus, to disrupt the target to the same amount with respect to its maximum achievable mass *M′*, collisions at *θ*_imp_ = 45° require roughly five times more energy, or around 2.2 times the impact velocity of those at *θ*_imp_ = 0° (since $E_{\mathrm{kin}}\propto \nu_{\mathrm{imp}}^{2}$). This result is intuitive as glancing angles poorly couple the impact energy to the target, and so this relation, at least in part, may be accounting for effects due to the interacting mass, albeit empirically. We do not explicitly model disruptive collisions at glancing angles, so the disruption threshold for glancing collisions, dictated by *α*, is often an extrapolation. Because embryo–embryo collisions beyond *ν*_imp_/*ν*_esc_ ~ 4 are rare, the fact that *α* is not precise for larger impact angles is not critical for modeling collisions in late-stage planet formation. It is also very important to acknowledge that Genda et al. (2015) found that the disruption threshold is not a converged quantity at resolutions beyond the state of the art in the literature (~10^6^ SPH nodes). Although they do not reach convergence at their highest resolution simulations, they find that the criterion is inversely proportional to the resolution in the target body, $K^{*}\propto n_{\mathrm{tar}}^{-1/3}$; our simulations, which have a range of target resolutions of (1–2) × 10^5^ nodes, are thus ~50%–60% higher than the expected value. In Figure 9, we show the 50% correction to the *α* relation.

In a hit-and-run collision, the mass is divided into three parcels: largest remnant, runner, and debris. To compute the mass of the runner, we first compute the maximum achievable mass of the runner,

(28) $$M_{\text{run}}^{\prime }=\{\begin{array}{ll} 0 & \text{not HnR}\\ M_{\text{tot}}-M_{\text{LR}}^{\prime }\equiv \xi_{\text{jump}}M_{\text{imp}} & \text{HnR}, \end{array}$$

where $M_{\text{LR}}^{\prime }$ is described by Equation (25). This formulation assumes that the amount of escaping debris just before the jump is equal to the amount of escaping debris just after the jump, at the onset of hit and run. The mass of the runner can be determined using a form similar to that of Equation (26),

(29) $$M_{\text{run}}=M_{\text{run}}^{\prime }\left(1-\frac{K}{\alpha U_{G}}\right).$$

We find that the value of *α* (see Equation (27)) fit for the disruption of the largest remnant provides a good fit to the mass of the runner for most cases. The mass of the escaping debris is computed using mass conservation:

(30) $$M_{\text{esc}}=\{\begin{array}{ll} M_{\text{tot}}-M_{\text{LR}} & \text{not HnR}\\ M_{\text{tot}}-M_{\text{LR}}-M_{\text{run}} & \text{HnR}. \end{array}$$

The residuals between the optimized model and the underlying data set for *M*_LR_, *M*_run_, and *M*_esc_ are shown in Figures 20, 21, and 22 and the associated figure sets.

## 6. Discussion

It is prudent to examine our model in the context of existing literature on the subject and describe its applicability to collisions outside the range of parameter space covered by our database. Because catastrophic disruption is a widely used concept in the giant-impact literature, this is a useful point in drawing comparisons. For the most complete scaling law for the mass of remnants in the literature, that of Leinhardt & Stewart (2012), we can also perform a complete comparison across our entire database.

### 6.1. Catastrophic Disruption Terminology

As an aside, we note that the term “catastrophic disruption” and its definition, as applied to giant impacts, is problematic and misleading. A clean (glancing) hit-and-run collision between two near-equal-mass bodies satisfies the *M*_LR_ = *M*_tot_/2 condition for catastrophic disruption; however, the target and impactor are somewhat intact. In this case, *M*_LR_ = *M*_tar_/2 more appropriately represents a “catastrophic” outcome, because in a clean hit and run, the target and impactor mass are almost entirely decoupled to begin with. Instead, we caution the use of “catastrophic disruption” terminology altogether, because of the risk of interpreting the threshold as one that is physical in nature, which is not the case in collisions of similar-sized bodies at probable impact geometries (grazing). However, we recognize its wide use as a benchmark across various studies, and it remains useful in head-on cases for similar-sized collisions.

### 6.2. Comparison of Catastrophic Disruption for Head-on, Equal-mass Collisions

Catastrophic disruption is only reached in our near-head-on collisions. For comparison to other work this is convenient because hit and run does not occur in head-on (*θ*_imp_ = 0) collisions; whether or not a scaling law predicts the onset of hit and run correctly is irrelevant here. We first consider collisions with *γ* = 1.

#### 6.2.1. Movshovitz et al. (2016)

**Figure 10.**
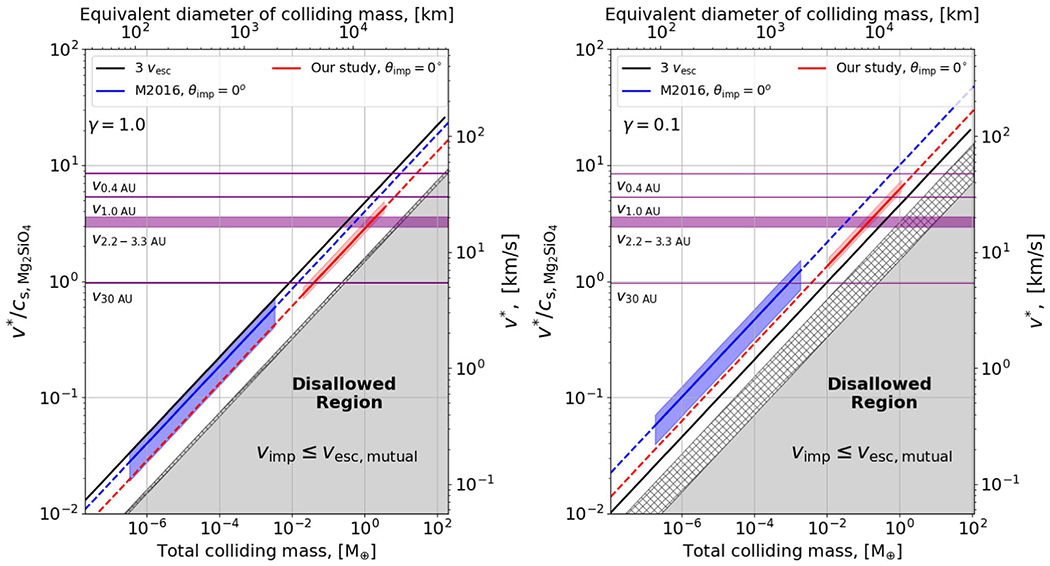
The same as Figure 1. but now includes our result for the disruption criteria for head-on collisions (red line). The red shaded band represents the entire range of disruption energies in our data; the red line represents the optimized value (*α*_0_/2 = 1.9). The blue line and shaded regions represent the range reported in Movshovitz et al. (2016). The transition to supersonic impact velocities occurs roughly at the boundary between the study of Movshovitz et al. (2016) and our study.

For Movshovitz et al. (2016), the comparison is straightforward because they use similar variables. They report that catastrophic disruption occurs at $K^{*}=(5.5\pm 2.9)U_{G}$. In our case for head-on collisions *α* = *α*_0_, and catastrophic disruption occurs at $K^{*}=\frac{1}{2}\alpha_{0}U_{G}$. From the MCMC optimization, we find $K^{*}=1.9U_{G}$; however, the individual values^11^ of $\frac{1}{2}\alpha_{0}$ span ~ 1.6–2.5. The catastrophic disruption energy found by Movshovitz et al. (2016) is thus about a factor of 2–3 higher than ours, with the upper and lower ranges being comparable, as shown in Figure 10. For equal-sized collisions (left panel), our head-on disruption energy is lower than the embryo collision velocities from Chambers (2013; black dotted line); thus, it is possible for large, equal-sized embryos to undergo some level of disruption, predicated on those collisions being nearly head on, which is a low-probability geometry (Shoemaker 1962). Even though our prediction is somewhat lower than that of Movshovitz et al. (2016), it is still very unlikely for collisions with *M*_tot_ ≈ 1 *M*_⊕_ and *γ* = 0.1 (right panel) to result in disruption at 1 au. Note also that Movshovitz et al. (2016) used significantly smaller bodies than our study, a point we consider further in Section 6.3.

#### 6.2.2. Stewart & Leinhardt (2009)

The comparison to Stewart & Leinhardt (2009) is somewhat more complicated because they use different variables, necessitating a conversion. In a head-on collision, $Q_{\mathrm{RD}}^{*}$ is the impact energy at catastrophic disruption, normalized by total mass, so

(31) $$Q_{\text{RD}}^{*}=\frac{1}{2}\frac{\alpha U_{G}^{*}}{M_{\text{tot}}},$$

Using $Q_{\mathrm{RD}}^{*}$ from Stewart & Leinhardt (2009) for equal-sized collisions (Equation (10)), we obtain

(32) $$\alpha R_{C1}^{2}=\frac{K^{*}}{M_{\text{tot}}}=\frac{1}{2}\frac{\alpha U_{G}}{M_{\text{tot}}}.$$

The value of *α* simplifies in the case of *θ*_imp_ = 0 (Equation (27)):

(33) $$\alpha R_{C1}^{2}=\frac{1}{2}\frac{\alpha_{0}U_{G}}{M_{\text{tot}}},$$

where $U_{G}$ is the numerically calculated binding energy of the whole system, because we use the numerical value to fit *α*. In the case of *γ* = 1, each body has a mass $\frac{1}{2}M_{\mathrm{tot}}$, and thus each has the same binding energy, $U_{G,\mathrm{tar}}=U_{G,\mathrm{imp}}$. Using the definition of Λ (Equation (18)), the total binding energy computed numerically, including the adjustment to account for the separation of the bodies (Equation (17)), is thus

(34) $$U_{G,\text{num}}=-\left(\frac{1}{8}+\frac{3}{10\Lambda }\right)\frac{GM_{\text{tot}}^{2}}{R},$$

where *R* is the radius of the target and impactor. For the small bodies of Stewart & Leinhardt (2009), we can reasonably assume that Λ = 1, and thus $U_{G}=\frac{17}{40}GM_{\text{tot}}^{2}/R$. Note that for our SiO_2_–Fe bodies Λ is typically around 0.91, which would make the prefactor closer to 18/40. It then follows that

(35) $$aR_{C1}^{2}=\frac{17}{60}\alpha_{0}\pi R^{2}\rho_{\text{bulk}}G,$$

where *ρ*_bulk_ is the bulk density of the body, which ranges from 500 to 3000 kg m^−3^ for the bodies in Stewart & Leinhardt (2009) and around 3000–5000 kg m^−3^ for our larger planets. Using the definition for *R*_C1_, we obtain

(36) $$a{\left(\frac{3}{4\pi }\frac{M_{\text{tot}}}{\rho {\text{H}}_{2}\text{O}}\right)}^{2/3}=\frac{17}{60}\alpha_{0}\pi R^{2}\rho_{\text{bulk}}G,$$

(37) $$a{\left(\frac{R^{2}\rho_{\text{bulk}}}{\rho {\text{H}}_{2}\text{O}}\right)}^{2/3}=\frac{17}{60}\alpha_{0}\pi R^{2}\rho_{\text{bulk}}G,$$

and the value of *a* can be solved for,

(38) $$a=\frac{17}{60}\alpha_{0}\pi G\rho_{\text{bulk}}^{1/3}\rho_{{\text{H}}_{\text{2}}\text{O}}^{2/3}.$$

Assuming a bulk density of 500–3000 kg m^−3^, as used by Stewart & Leinhardt (2009), and *±*_0_ ≈ 3.75 this gives *a* ≈ (1.7–3.2) × 10^−8^ m kg^−1/3^ s^−2^. For densities more relevant to our planets, ~3000–5000 kg m^−3^, the range is *a* ≈ (3.2–3.8) × 10^−8^ m kg^−1^/^3^ s^−2^. In the cgs units used by Stewart & Leinhardt (2009), these ranges become *a* ≈ (1.7–3.2) × 10^−7^ cm g^−1/3^ s^−2^ and *a* ≈ (3.2–3.8) × 10^−7^ cm g^−1/3^ s^−2^. Thus, our value is within a factor of 3–10 of the *a* = (1.7 ± 0.3) × 10^−6^ cm^2^ g^−2/3^ s^−2^ reported by Stewart & Leinhardt (2009). In this case, our disruption criterion is lower; however, Stewart & Leinhardt (2009) used much smaller bodies than our study. Again, this is a point we consider further in Section 6.3.

#### 6.2.3. Leinhardt & Stewart (2012)

As done for Stewart & Leinhardt (2009), we must convert to the different formulation used by Leinhardt & Stewart (2012). We begin by again equating $Q_{\mathrm{RD}}^{*}$ and K*/*M*_tot_, this time using the relation for catastrophic disruption of Leinhardt & Stewart (2012) at *γ* = 1 (Equation (13)), from which we obtain

(39) $$c^{*}\frac{4}{5}\pi \rho_{{\text{H}}_{\text{2}}\text{O}}GR_{\mathrm{RC}1}^{2}=\frac{1}{2}\frac{\alpha_{0}U_{G}M_{\text{tot}}}{R}.$$

As before, we then use $U_{G}=\frac{17}{40}GM_{\text{tot}}^{2}/R$, which gives us

(40) $$c^{*}R_{\text{C}1}^{*}=\frac{17}{48}\alpha_{0}R^{2}\frac{\rho_{\text{bulk}}}{\rho_{{\text{H}}_{\text{2}}\text{O}}},$$

and substituting in for *R*_C1_,

(41) $$c^{*}=\frac{17}{48}\alpha_{0}{\left(\frac{\rho_{\text{bulk}}}{\rho_{{\text{H}}_{\text{2}}\text{O}}}\right)}^{\frac{1}{3}}.$$

In this case, c* ≈ 1.9–2.3 for bulk densities of 3000–5000 kg m^−3^ and if *α*_0_ = 3.75. Leinhardt & Stewart (2012) report two values of c*, the relevant one being that which was fit to collisions between hydrodynamic planets in the literature, c* = 1.9 ± 0.3, which is very close to our value. A value of c* = (5 ± 2) was also reported for smaller bodies, some of which were modeled with strength while others only featured self-gravity. Nevertheless, this number is close to the values found by Stewart & Leinhardt (2009) and Movshovitz et al. (2016), implying there may exist some dependence of the catastrophic disruption threshold on the scale (total mass) of the collision.

### 6.3. Scale Dependence

Gravity-dominated collisions modeled in our study and in previous work span several orders of magnitude in *M*_tot_. from Earth-mass planets down to bodies tens of kilometers in size. So, it is prudent to address whether scale effects exist within the pure-gravity regime. As described in Asphaug (2010) and discussed in Leinhardt & Stewart (2012), detailed impact outcomes, such as the irreversible increases in entropy due to shock heating, production of vapor, etc., will undoubtedly depend on the scale and thus absolute velocity of the collision. For example. Burger et al. (2017) find vapor production to be strongly governed by the scale (absolute velocity) of the collision. However, here we are concerned with whether these thermodynamic effects may be driving changes in the bulk outcomes (mass of remnants), which are assumed to be invariant of scale in the literature thus far. We also distinguish scale dependence in the gravity regime from those that are well documented near the strength regime (at smaller scales), which is discussed in Section 3.1.

We noted in Section 6.2 that our value for the catastrophic disruption energy for a head-on, *γ* = 1 collision matched well with what Leinhardt & Stewart (2012) found for large. hydrodynamic planets. However, the values found by Stewart & Leinhardt (2009) and Movshovitz et al. (2016) for smaller gravity-dominated bodies were consistently higher. For example, Movshovitz et al. (2016) used hydrodynamic bodies (modeled with the Tillotson equation of state) with masses of 10^−6^–10^−3^ *M*_⊕_, one to four orders of magnitude smaller in mass than ours. The fitted relation from Stewart & Leinhardt (2009) for subsonic collisions between gravity-dominated rubble-pile aggregates, the relation we examine herein (Equation (10)), involved even less massive bodies. This suggests that some amount of scale dependence may exist, where smaller gravity-dominated bodies require more energy to disrupt, relative to their binding energy, than bodies greater than ~10^−3^–10^2^*M*_⊕_.

A possible source for the difference in scaling laws in the pure-gravity regime is the transition from subsonic to supersonic collisions. In Figure 10, we show the scaling laws of Movshovitz et al. (2016) and ours, with the solid lines representing the range of bodies simulated in the respective works. The *ν*_30 au_ line indicates roughly the threshold for subsonic to supersonic collisions (ν*/*c*_s,Mg_2_SiO_4__ ≈ 1). It is particularly striking that this transition occurs roughly at the boundary between our study and that of Movshovitz et al. (2016), indicating potential scale-dependent effects within the pure-gravity regime, due to the onset of shock-generating collisions. This result would be counter to the classical argument developed for the context of cratering collisions, that supersonic collisions less efficiently translate impact energy into kinetic energy (or excavation) of the target medium, due to the production of “waste heat” (Holsapple 1993).

It is important to note that numerical effects also play a role in the estimation of the disruption energy (Genda et al. 2015). The disruption threshold is artificially greater in lower resolution simulations, and results at lower resolutions can be scaled according to the target resolution $K^{*}\propto n_{\mathrm{tar}}^{1/3}$ (Genda et al. 2015). Thus, the 5 × 10^4^ node simulations in Movshovitz et al. (2016) should have a disruption threshold ~25% lower at an equivalent resolution to our simulations performed with *n*_tar_ = 1 × 10^5^ nodes, shrinking the discrepancy between the two studies, but not resolving it entirely. Whether $U_{G}$ is computed numerically or analytically (using the constant-density approximation) is also an important consideration, particularly when the colliding bodies have differentiated structures or are large in scale. Due to these effects the difference between the true value and the approximation is ~1%–20% (see Table 1), enhancing the discrepancy between the two studies.

We must also bear in mind our limited ability to directly compare differences in thermodynamically driven effects between studies in the literature that use different numerical schemes, different equations of state that may not be thermodynamically consistent (e.g., Tillotson in Movshovitz et al. 2016; Burger et al. 2017) or initial thermal conditions. We also emphasize the weakness of this dependence, if it is present: for example, our smallest targets are 10^7^ times more massive than the targets used by Stewart & Leinhardt (2009) and yet the difference in the disruption criterion for their small bodies is a factor of ~3–10. In order to address whether scale dependence exists (in terms of remnant masses), due to transition to supersonic impact velocities, one must carefully consider the equation of state in use across the different studies, as the thermodynamic response of materials in the giant-impact literature are not directly comparable. For these reasons, evidence for scale dependence is limited at this time and thermodynamic arguments (waste heating) would indicate trends opposite those reported in this section.

### 6.4. Comparison of Catastrophic Disruption in the Limit of Small Impactors

It is also prudent to examine our empirical model in the limit of small impactors (*γ* → 0), in comparison with those of previous work. Note that we do not encourage the use of any giant-impact scaling laws in the cratering regime (*γ* → 0 with impact energies well below disruption), and accuracy cannot be guaranteed in *γ* → 0 cases generally, because the physics of the former scenario are of a different scale than the SPH simulations used in giant-impact literature. Nonetheless, this limit is still useful for examining the behavior of the scaling relationships.

Per Equation (27), as *γ* → 0, *θ*_imp_ dependence on the disruption energy vanishes and *α* → *α*_0_ = 3.75. This implies that as *γ* → 0, the efficiency with which the impact energy is coupled into the target is independent of *θ*_imp_, but is not entirely independent of *γ* as $U_{G}$ includes a contribution from the impactor (see Equation (17)). Physically, the disappearance of dependence on *θ*_imp_ at very small *γ* is somewhat intuitive; over the range of *θ*_imp_, either all of the impactor will strike the target or all of the impactor will miss the target. Furthermore, at very small *γ*, impacts with energies significantly lower than required for disruption can be well modeled as striking an infinite plane. However, it is also reasonable to expect that impact energy will be more poorly coupled to the target in off-axis collisions, such as in the case of scouring collisions (e.g., Schultz & Wrobel 2012); however, this regime is not covered in our simulations of giant impacts.

The indirect dependence of *γ* in Movshovitz et al. (2016), due to the interacting mass, disappears for head-on collisions because the interacting mass is equal to the total mass in that case. Their scaling law thus similarly trends to a constant value as *γ* → 0. This is significant as their collisions includes a large range of impactor-to-target mass ratios, from *γ* = 1 to *γ* ≈ 0.01.

Differing from the others, Leinhardt & Stewart (2012) cast their scaling laws in terms of a fit for $Q_{\text{RD}}^{*}$ at *γ* = 1 with a correction for scenarios with *γ* < 1,

(42) $$Q_{\text{RD}}^{*}=Q_{\text{RD,}\gamma \text{=1}}^{*}{\left(\frac{1}{4}\frac{{\left(\gamma +1\right)}^{2}}{\gamma }\right)}^{2/3\bar{\mu }-1}.$$

They fit $\bar{\mu }=0.35$, such that the index $2/(3\bar{\mu })-1\approx 0.9$, and thus in the limit *γ* → 0, $Q_{\text{RD}}^{*}\to \infty$. This implies that very small impactors cannot disrupt the target, regardless of impact energy, which we find unphysical.

Benz & Asphaug (1999) demonstrated that the disruption threshold for off-axis collisions is systematically higher across the entire study; however, at each angle a new impactor size (or equivalently *γ*) was determined. Because *γ* was not held constant or reported, the dependence of $Q_{\text{D}}^{*}$ on *γ* is unclear, and indeed. Equation (4) does not have direct dependence on *γ*. However, given that $Q_{\text{D}}^{*}$ is clearly dependent on *θ*_imp_ across their study, it may be the case that in the strength-dominated regime, dependence on *θ*_imp_ in the limit of *γ* → 0 exists.

### 6.5. Full Comparison to the Leinhardt & Stewart (2012) Formalism

We can also perform a comparison to the full scaling law formalism of Leinhardt & Stewart (2012), which is the most extensive of previous scaling law efforts for giant impacts. We use the value of c* = 1.9 ± 0.3 suggested by Leinhardt & Stewart (2012) for large hydrodynamic planets. We first performed several tests to ensure our implementation of their model is accurate considering the many steps involved. This includes ensuring the value of the specific impact energy and interacting mass fraction reported in their Table 2 matches our implementation. When using their catastrophic disruption energy $Q_{\text{RD}}^{\prime *}$ the masses of the largest and second largest remnants agree between our implementation and their Table 2.

**Figure 11.**
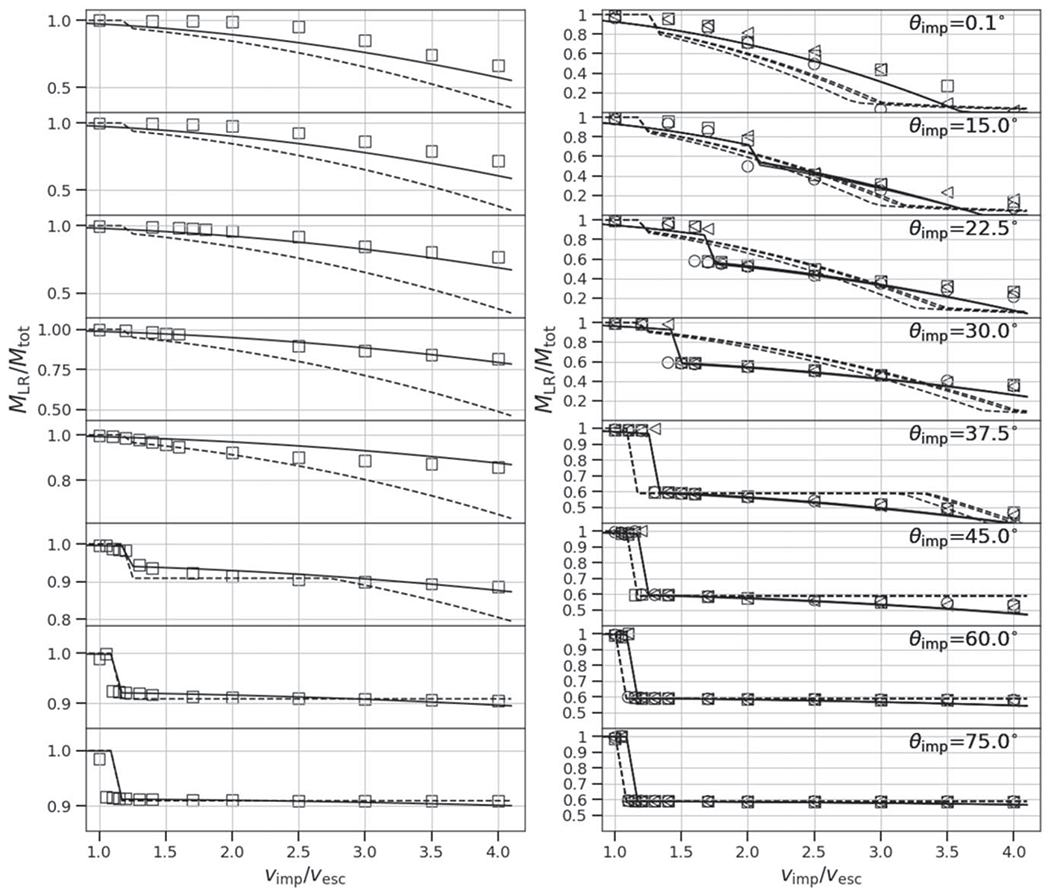
The largest remnant mass, normalized by the total mass, for the giant-impact simulations of differentiated, two-layer, SiO_2_–Fe planets with an impactor-to-target mass ratio of *γ* = 0.1 (left) with *M*_tar_ = 0.1 *M*_⊕_ and *γ* = 0.7 (right) with *M*_tar_ = 1.0 *M*_⊕_, 0.1 *M*_⊕_, and 0.01 *M*_⊕_. Circle, square, and triangle symbols represent data for *M*_tar_ = 1.0 *M*_⊕_, 0.1 *M*_⊕_, and 0.01 *M*_⊕_, respectively. The solid line represents the prediction from our model and the dashed line represents that of Leinhardt & Stewart (2012). Both scaling laws predict the hit-and-run transition in the *γ* = 0.1 case, which is demonstrated by the discontinuity at *γ*_imp_ = 45°. At larger *γ* (right), the grazing criterion (Asphaug 2010) implemented by Leinhardt & Stewart (2012) does not predict the existence of hit-and-run collisions in the $\theta_{\mathrm{imp}}>22_{.}^{{}^{\circ}}5$ and 30° data.

As shown in Figure 11, both our predictions and those of Leinhardt & Stewart (2012) match our data well at *γ* = 0.1. For larger impactor-to-target mass ratios, the predictions of Leinhardt & Stewart (2012) less accurately predict the transition to hit and run. This is illustrated in the right-hand panels of Figure 11 for the $\theta_{\mathrm{imp}}=22_{.}^{{}^{\circ}}5$ and 30° cases. At $\theta_{\mathrm{imp}}=37_{.}^{{}^{\circ}}5$, Leinhardt & Stewart (2012) correctly predicted that hit and run occurs; however, it predicts the onset of hit and run at a lower velocity than seen in our simulation data and predicts a constant largest remnant mass until the onset of erosion at *ν*_imp_ ~ 3.25*ν*_esc_, whereas our model includes the erosion of the target in hit-and-run scenarios.

The prediction of hit and run at lower angles in our work is expected as generally, our geometric criterion for hit and run (Equation (21)) is lower than the grazing angle condition, which was not intended to be a hit-and-run criterion (Asphaug 2010). Moreover, this underprediction of hit and run in models that implement the simple grazing angle criterion from Asphaug (2010) grows for collisions between bodies of greater density stratification. This suggests that the number of hit-and-run collisions reported by Chambers (2013; ~42% for embryo–embryo collisions) and by Kokubo & Genda (2010; ~49% in simulations that involved strictly embryos) are lower bounds on the prevalence of hit and run, but may be overestimates for the lowest velocity collisions. However, because the impact parameters (particularly *γ* for each of the collisions) in those *N*-body studies are not reported, we cannot estimate the fraction of merging or erosive collisions that would be predicted as hit and run by our model. Furthermore, as demonstrated in Chambers (2013) and Emsenhuber & Asphaug (2019), a sequence of multiple hit-and-run collisions between two bodies can occur, so it is reasonable to expect many more hit-and-run collisions from *N*-body codes that implement our geometric criterion.

**Figure 12.**
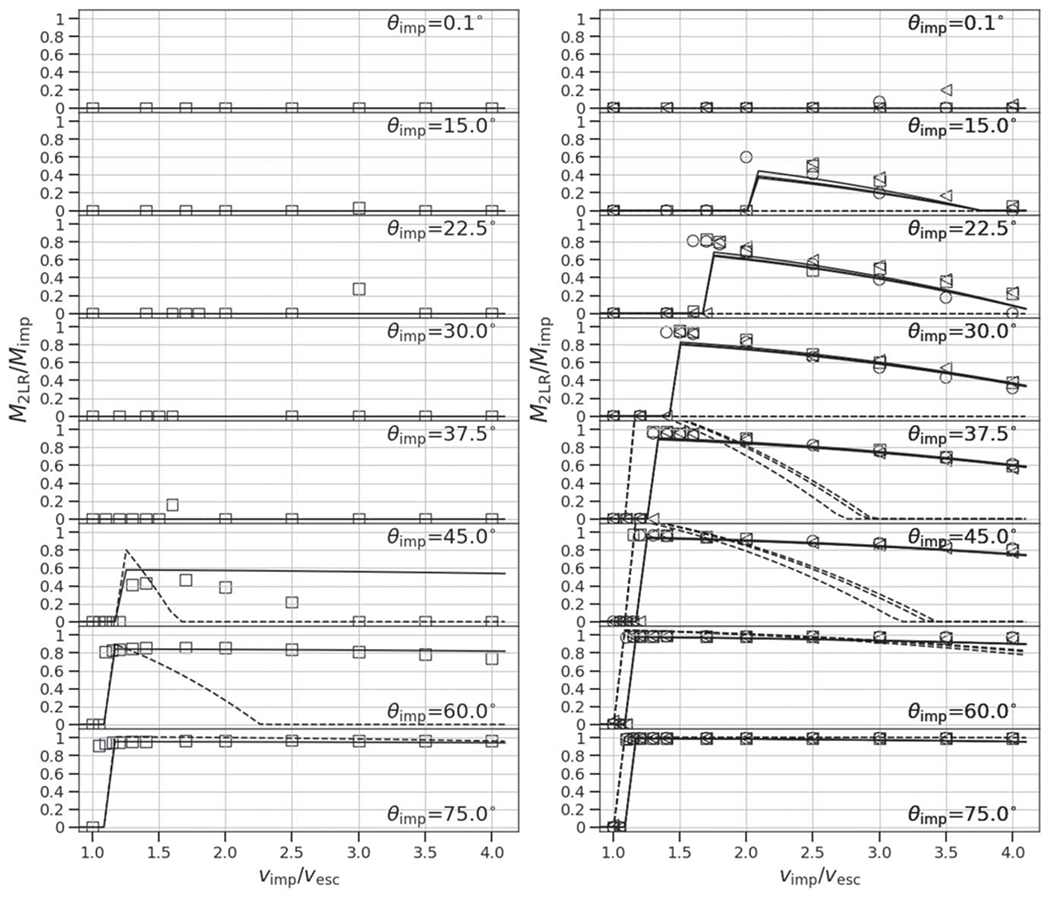
The second largest remnant mass, normalized by the impactor mass, for the giant-impact simulations of differentiated, two-layer, SiO_2_–Fe planets with an impactor-to-target mass ratio of *γ* = 0.1 (left) with *M*_tar_ = 0.1 *M*_⊕_ and *γ* = 0.7 (right) with *M*_tar_ = 1.0 *M*_⊕_, 0.1 *M*_⊕_, and 0.01 *M*_⊕_. Circle, square, and triangle symbols represent data for *M*_tar_ = 1.0 *M*_⊕_, 0.1 *M*_⊕_, and 0.01 *M*_⊕_, respectively. The solid line represents the prediction from our model and the dashed line represents that of Leinhardt & Stewart (2012).

In terms of the mass of the runner, we generally find both scaling laws have issues for hit-and-run collisions at low *γ*. For example, in the left-hand panel of Figure 12, both scaling laws predict hit and run for *θ*_imp_ = 45°, but the scaling law of Leinhardt & Stewart (2012) overpredicts the amount of erosion of the projectile (smaller runner) whereas our relation underpredicts the erosion of the runner. However, we find that at larger angles, our scaling law agrees well with the simulation data, as seen in the *θ*_imp_ = 60° case. At larger *γ* (see the right-hand panel of Figure 12), we find the scaling laws of Leinhardt & Stewart (2012) significantly overpredict the level of erosion in the runner at *ν*_imp_ > 2*ν*_esc_, as can be seen in the $\theta_{\mathrm{imp}}=37_{.}^{{}^{\circ}}5$ and 45° cases; however, this discrepancy diminishes at more grazing angles.

**Figure 13.**
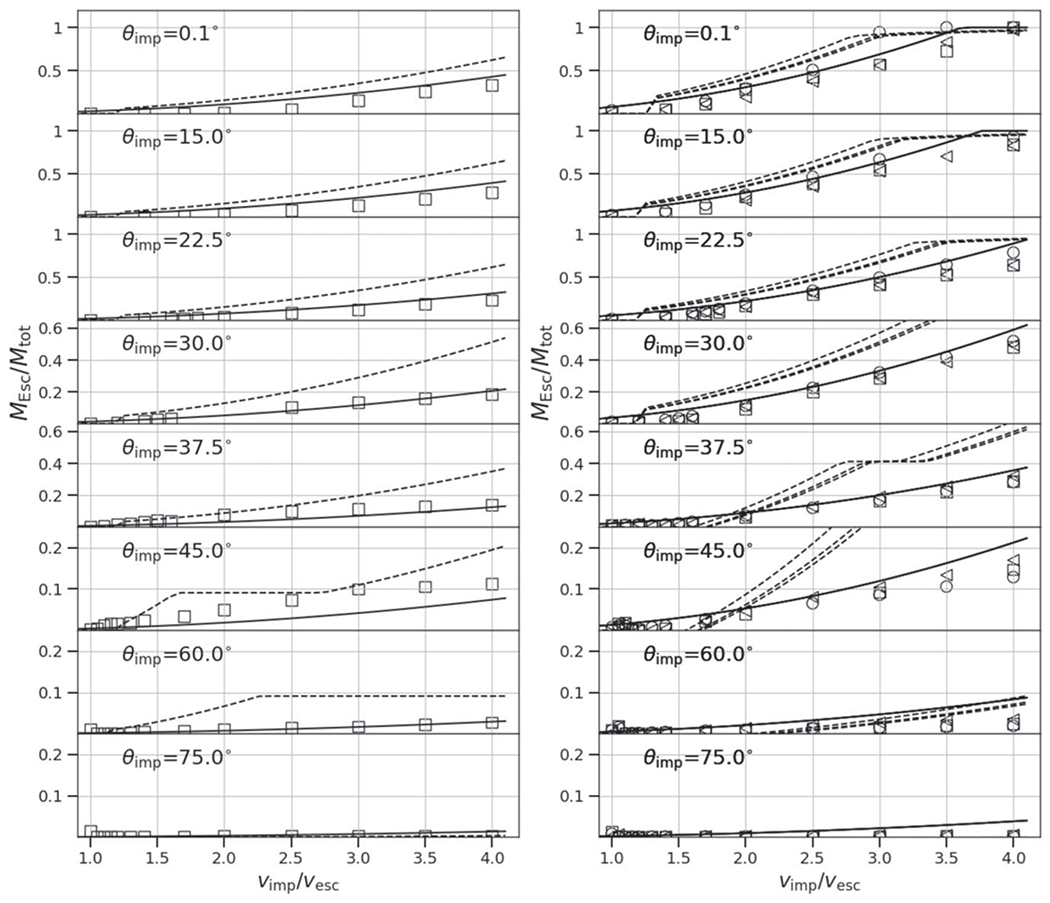
The escaping mass, normalized by the total colliding mass, for the giant-impact simulations of differentiated, two-layer, SiO_2_–Fe planets with an impactor-to-target mass ratio of *γ* = 0.1 (left) with *M*_tar_ = 0.1 *M*_⊕_ and *γ* = 0.7 (right) with *M*_tar_ = 1.0 *M*_⊕_, 0.1 *M*_⊕_, and 0.01 *M*_⊕_. Circle, square, and triangle symbols represent data for *M*_tar_ = 1.0 *M*_⊕_, 0.1 *M*_⊕_, and 0.01 *M*_⊕_, respectively. The solid line represents the prediction from our model, and the dashed line represents that of Leinhardt & Stewart (2012).

**Figure 17.**
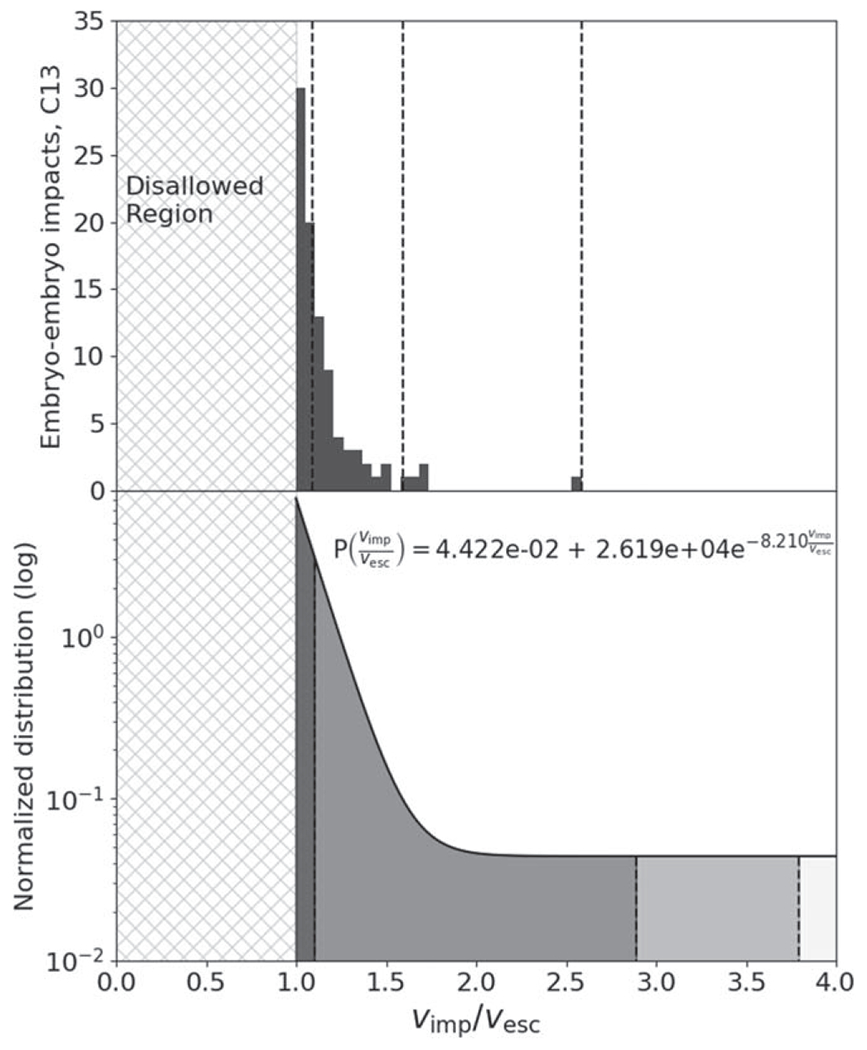
Impact velocity distribution for embryo–embryo collisions in Chambers (2013; their Figure 6). The top panel shows a histogram of the raw values and the bottom panel shows a fitted probability distribution that is truncated at 4*ν*_esc_ and normalized to an area of unity. The three dashed lines represent the 50th, 95th, and 99th percentiles.

**Figure 20.**
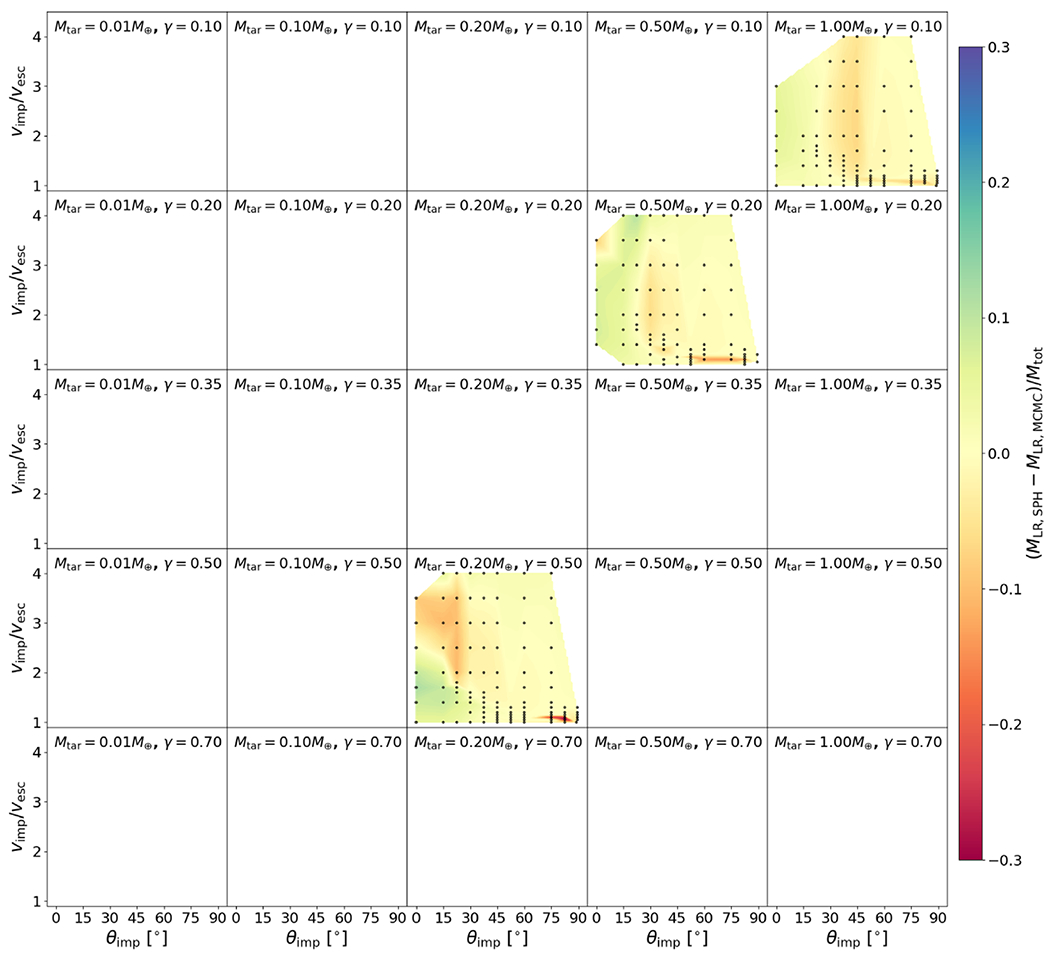
The deviations in *M*_LR_/*M*_tot_ for the pure SiO_2_ bodies between the prediction of the best-fit model provided by the MCMC method herein and the simulated data on which the model was fit. Each panel represents a unique combination of impactor-to-target mass ratio (*γ*; rows) and target mass (*M*_tar_; columns). Lighter colors represent minimal deviation of the MCMC-optimized model from the SPH collision data. (The complete figure set (3 images) is available.)

**Figure 21.**
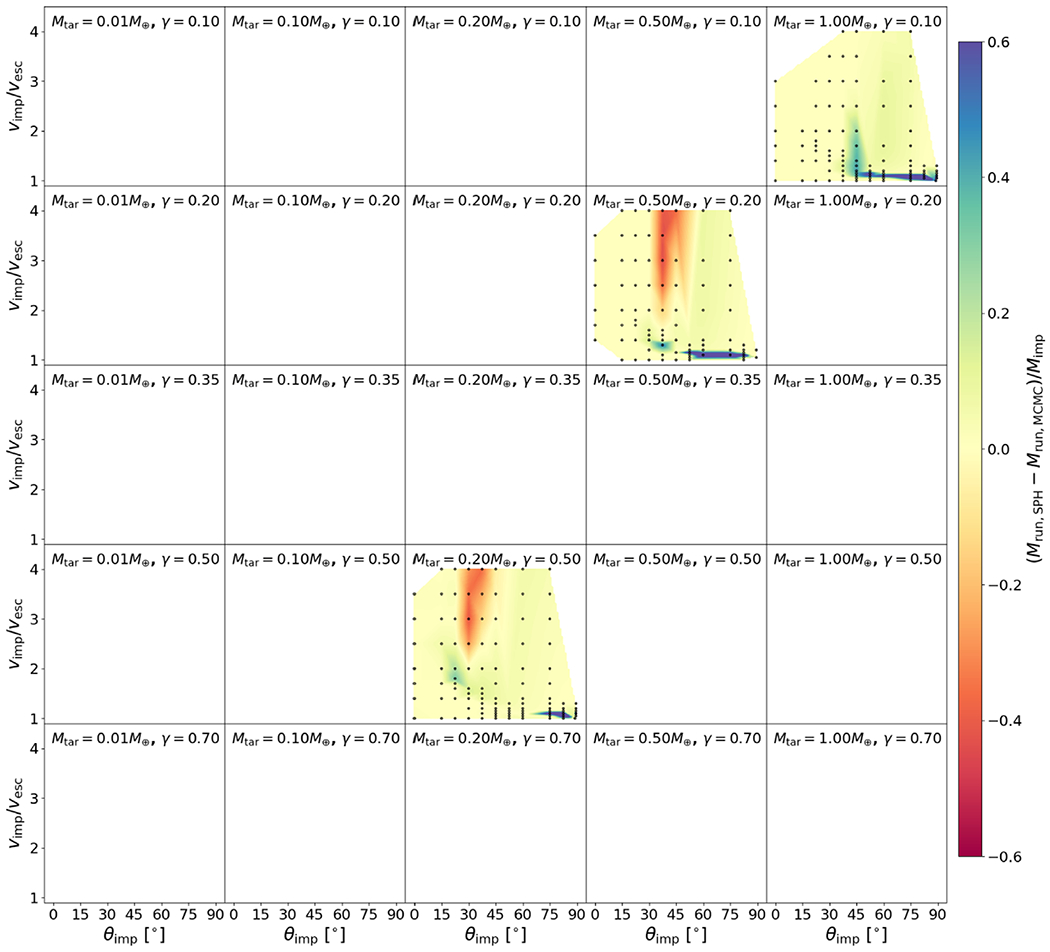
The deviations in *M*_run_/*M*_imp_ for the pure SiO_2_ bodies between the prediction of the best-fit model provided by the MCMC method herein and the simulated data on which the model was fit. Each panel represents a unique combination of impactor-to-target mass ratio (*γ*; rows) and target mass (*M*_tar_; columns). Lighter colors represent minimal deviation of the MCMC-optimized model from the SPH collision data. (The complete figure set (3 images) is available.)

**Figure 22.**
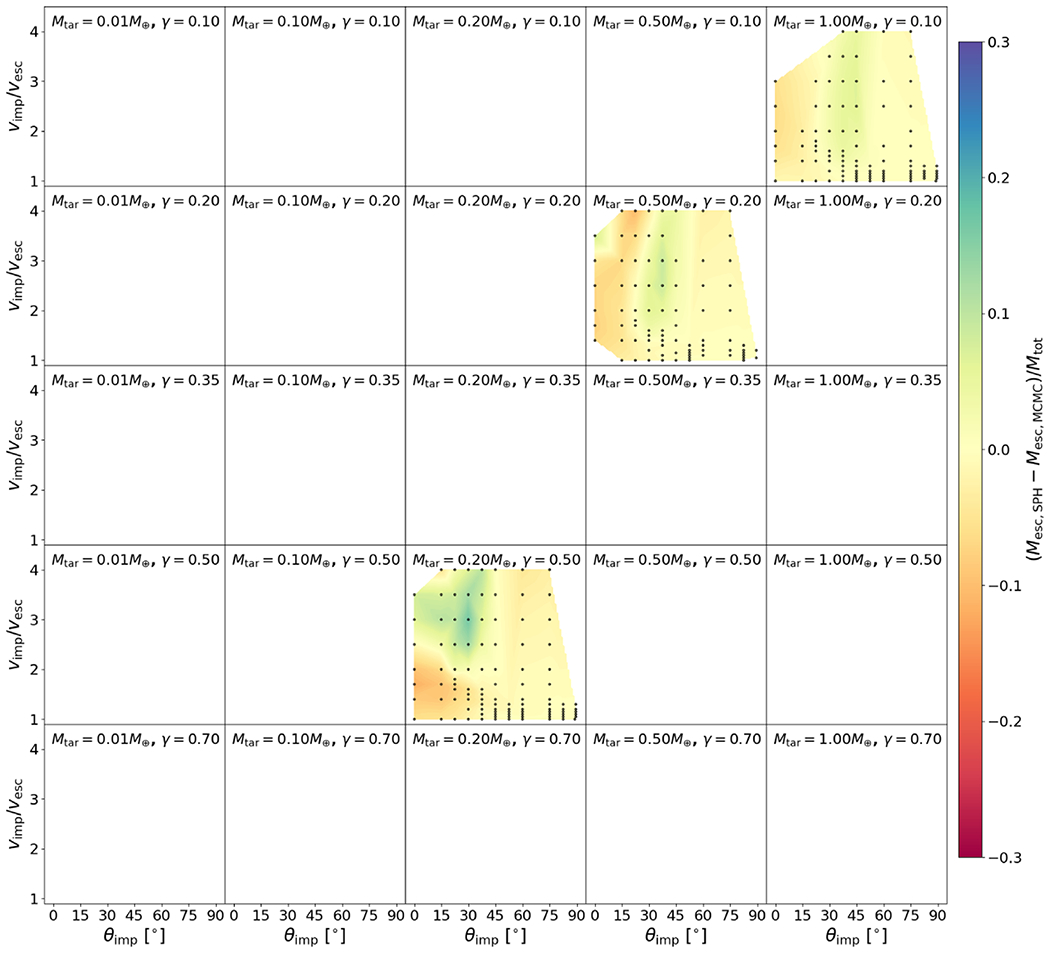
The deviations in *M*_esc_/*M*_tot_ for the pure SiO_2_ bodies between the prediction of the best-fit model provided by the MCMC method herein and the simulated data on which the model was fit. Each panel represents a unique combination of impactor-to-target mass ratio (*γ*; rows) and target mass (*M*_tar_; columns). Lighter colors represent minimal deviation of the MCMC-optimized model from the SPH collision data. (The complete figure set (3 images) is available.)

Under our formalism, the runner is always less massive than the projectile because *ξ*_jump_ is less than 1 (see Equation (24)). This is not always the case in the formalism of Leinhardt & Stewart (2012), where at low velocities the runner can be more massive than the projectile,^12^ a phenomenon not observed in our simulations. As for the prediction of escaping mass, in both our framework and that of Leinhardt & Stewart (2012), mass is conserved such that *M*_tot_ = *M*_LR_ + *M*_esc_ for non-hit-and-run collisions or *M*_tot_ = *M*_LR_ + *M*_run_ + *M*_esc_ in the case of hit and run, where *M*_run_ is the mass of the impactor after the collision. Therefore, where there is a discrepancy in the prediction of the mass of the largest remnants, there is also a discrepancy in the predicted escaping mass (see Figure 13).

In Appendices D–F, we provide a comparison of the prediction of *M*_LR_ between the two models against three distinct data sets: the disruptive collisions in Movshovitz et al. (2016), the data set herein, and the PKDGRAV simulation data from Leinhardt & Stewart (2012; their Table 4). Note that in our model we do not attempt to describe the size distribution of smaller remnants. This is not resolved in SPH simulations of giant impacts at our resolution, which is why we have limited this analysis to the total target, runner, and debris masses. Users of our algorithm in the next section should refer to Leinhardt & Stewart (2012) and others if they require estimates for the size-frequency distribution of debris. For the realms of giant impacts we have considered, we find that our model and that of Leinhardt & Stewart (2012) show residuals centered about zero with minimal systematic bias and a low mean squared error (MSE), which is a measure of the accuracy of the model across the data set. Our model has, at worst, an MSE of ~0.08. This corresponds to an expected residual in *M*_LR_ values of 0.28 *M*_tot_ for “supercatastrophic” scenarios, and an expected residual of 0.07 *M*_tot_ across our database, which emphasizes the range of collision velocities and geometries expected in late-stage planet formation. We find that the MSE of the Leinhardt & Stewart (2012) model is often greater, and in most cases the discrepancy increases when adjusting for the probability distribution of impact angles (*P*(*θ*_imp_) = sin(2*θ*_imp_)). As noted, some of this discrepancy is due to the underprediction of hit and run when using the Asphaug (2010) grazing criterion. It is equally important to also consider the inherent deviation of SPH simulation results, due to differences in methodology and initial conditions, which should temper the tendency to reproduce exactly the same results from any single simulation. For example, thermal conditions (e.g., Genda et al. 2015), pre-impact rotation (e.g., Canup 2008; Rufu et al. 2017), resolution (e.g., Genda et al. 2012), and the choice of artificial viscosity (e.g., Hosono et al. 2019) are all known to affect the impact outcome, not just in terms of the thermodynamic end state but sometimes in terms of the occurrence of hit and run, the masses of remnants, etc. To know the mass of remnants to within very high precision, one must take into consideration, at a minimum, resolution convergence, as we do in Section 6.3, to estimate the true value (as opposed to that provided by simulations); still, only for the specific thermal and rotational state that was simulated is the result accurate, assuming the choice of SPH kernel and artificial viscosity is appropriate.

## 7. Prescription for *N*-body Codes

Below we provide a step-by-step methodology for estimating the outcome of a gravity-dominated collision in an *N*-body environment.

1. Determine *M*_tar_ and *M*_imp_ such that *M*_tar_ ⩾ *M*_imp_. Determine *θ*_imp_ and *ν*_imp_ from the relative position and velocity vectors, and compute *M*_tot_ = *M*_tar_ + *M*_imp_, *μ* = *M*_tar_*M*_imp_/*M*_tot_, *γ* = *M*_imp_/*M*_tar_, $\nu_{\mathrm{esc}}=\sqrt{2GM_{\mathrm{tot}}/(R_{\mathrm{imp}}+R_{\mathrm{tar}})}$, and $K=\frac{1}{2}\mu \nu_{\mathrm{imp}}^{2}$.
2. Compute the gravitational binding energy analytically (Equations (16) and (17)) and numerically (Equation (15)) to determine Λ (Equation (18)). If the density structure is not tracked, use Λ ≈ 0.88 and 0.95 for differentiated water-rich and rocky planets, respectively. For stripped cores or large homogeneous bodies, use ~0.98. In the limit of small homogeneous bodies, use Λ = 1.
3. Determine if *θ*_imp_ > *θ*_HnR_ and *ν*_imp_/*ν*_esc_ > *ν*_HnR_, where *θ*_HnR_ = −33.8 log_10_(*γ*) + 20.6Λ^8.9^ and *ν*_HnR_ = 1.88(1 − sin(*θ*_imp_))^5/2^ + 1.13.
  - If not, the collision is not hit and run, and $M_{\mathrm{LR}}^{\prime }=M_{\mathrm{tot}}$.
  - If so, compute $\xi_{\text{jump}}=1-\frac{1}{2}{\left(\frac{\theta_{\text{HnR}}\left(90^{\circ }-\theta_{\text{imp}}\right)}{\theta_{\text{imp}}\left(90^{\circ }-\theta_{\text{HnR}}\right)}\right)}^{1.42}$, then compute the maximum achievable mass of the largest remnant for the hit-and-run case: $M_{\mathrm{LR}}^{\prime }=M_{\mathrm{tot}}-\xi_{\mathrm{jump}}M_{\mathrm{imp}}$.
4. Compute $\alpha 10^{-4.90}\theta_{\mathrm{imp}}^{3.72}+3.75$, and if the total mass is less than ~0.01 *M*_⊕_, then an additional factor of ~2.8 on *α* may be used to match results from Movshovitz et al. (2016). To approximate the true erosion expected in a collision, reduce the value of *α* by 50% (Genda et al. 2015).
5. Compute mass of the largest remnant: $M_{\mathrm{LR}}=\mathrm{MAX}\;(0,M_{\mathrm{LR}}^{\prime }(1-K/\alpha U_{G}))$.
6. If collision was hit and run, compute $M_{\mathrm{run}}^{\prime }=\xi_{\mathrm{jump}}M_{\mathrm{imp}}$, then compute the mass of the runner: $M_{\mathrm{run}}=\mathrm{MAX}\;(0,M_{\mathrm{run}}^{\prime }(1-K/K^{*}))$.
7. Compute *M*_esc_ = *M*_tot_ − *M*_LR_ − *M*_run_.

## 8. Conclusions

We have developed a model that accurately predicts the mass of remnants in giant impacts between gravity-dominated bodies and can be easily adopted into *N*-body methods. Using an MCMC method, the model was optimized to results from over ~ 1400 SPH simulations that span the most relevant conditions expected in late stages of planet formation. A weighted MCMC scheme was used to globally optimize the model to the entire data set and account for imbalances in the simulated impact conditions.

Because roughly half of the time a population of self-stirred bodies is expected to produce hit-and-run events, where only a portion of the impactor and target directly intersect, and the impactor continues downrange in a deflected trajectory (e.g., Agnor & Asphaug 2004), we pay particular attention to this regime. The transition of collisions from merging to hit and run occurs as a fairly sharp boundary in the pre-impact parameter space, namely at low velocities and grazing angles, so we finely sampled the parameter space in these regions. This allowed for the development of a greatly improved hit-and-run criterion and thus a more accurate prediction of the masses of remnants compared to the prevailing models in the literature.

By modeling planets of variable composition, we found that the density stratification of the colliding bodies leads to hit-and-run collisions at lower angles than suggested by using the grazing rule (Asphaug 2010). Considering this, we expect that primitive (undifferentiated) bodies early in the formation process and stripped cores late in the formation process (or in dynamically stirred regions) may undergo hit and run least often. Collisions between differentiated bodies will be hit and run more often, increasing the accretion timescale. This effect demonstrates that the accretion dynamics and timescales are contingent on the internal structure of the planets in the dynamical environment, an aspect not currently accounted for in state-of-the-art *N*-body planet formation codes. We also demonstrate that hit-and-run collisions do not result in the target and impactor emerging unscathed, with no erosion of either body, but rather exhibit a range of accretion efficiencies that is dependent on the impactor-to-target mass ratio and impact angle (e.g., Agnor & Asphaug 2004), a behavior we fit empirically.

We also report a potential transition within the pure-gravity regime that violates the commonly assumed scale-invariance assumption for giant impacts. Comparisons with other studies suggest erosion may be enhanced in collisions where the impact velocity is supersonic, which occurs in a self-stirred system of planetary embryos with masses ~ 10^−3^–10^−2^*M*_⊕_. In light of our results, it is also reasonable to expect that a complicated interplay of thermodynamic effects and density stratification governs the onset of hit and run and other impact outcomes. The onset of differentiation, which occurs early on due to heating from ^26^A1 heating, and the occurrence of supersonic collisions between self-stirred embryos, which occurs later when embryos reach masses roughly that of Earth’s Moon, mark two new potential transitions in the process of planet formation.

We also argue that the commonly used definition of the catastrophic disruption threshold energy, the impact energy at which the largest remnant has half of the total mass after collision, is inappropriate in the case of collisions between similar-sized bodies of any scale. A hit-and-run scenario between two equal-mass bodies produces a largest remnant that has half the total mass after collision (satisfying the catastrophic disruption criteria), but it may be minimally disrupted. In this case, the morphological result of the collision would not reflect the disruption of either body and certainly could not be described as “catastrophic.” We insist that catastrophic disruption generally does not describe the outcome of collisions between major bodies during terrestrial planet formation and is a metric that is useful only when the bodies are disparately sized or in head-on scenarios.

Finally, our empirical model for estimating giant-impact outcomes is readily implemented into *N*-body codes, allowing them to track the mass (and by mass loss or gain, the composition) of large remnants and debris after a collision in the purely gravity-dominated regime. We provide adjustments for the possible phenomenon of scale dependence in the gravity regime as well as numerical convergence effects expected from the resolution of our simulations.

## Supplementary Material

Full-length version of Table 3

Full-length version of Table 5

Full-length version of Table 4

## Figures and Tables

**Figure 14.**
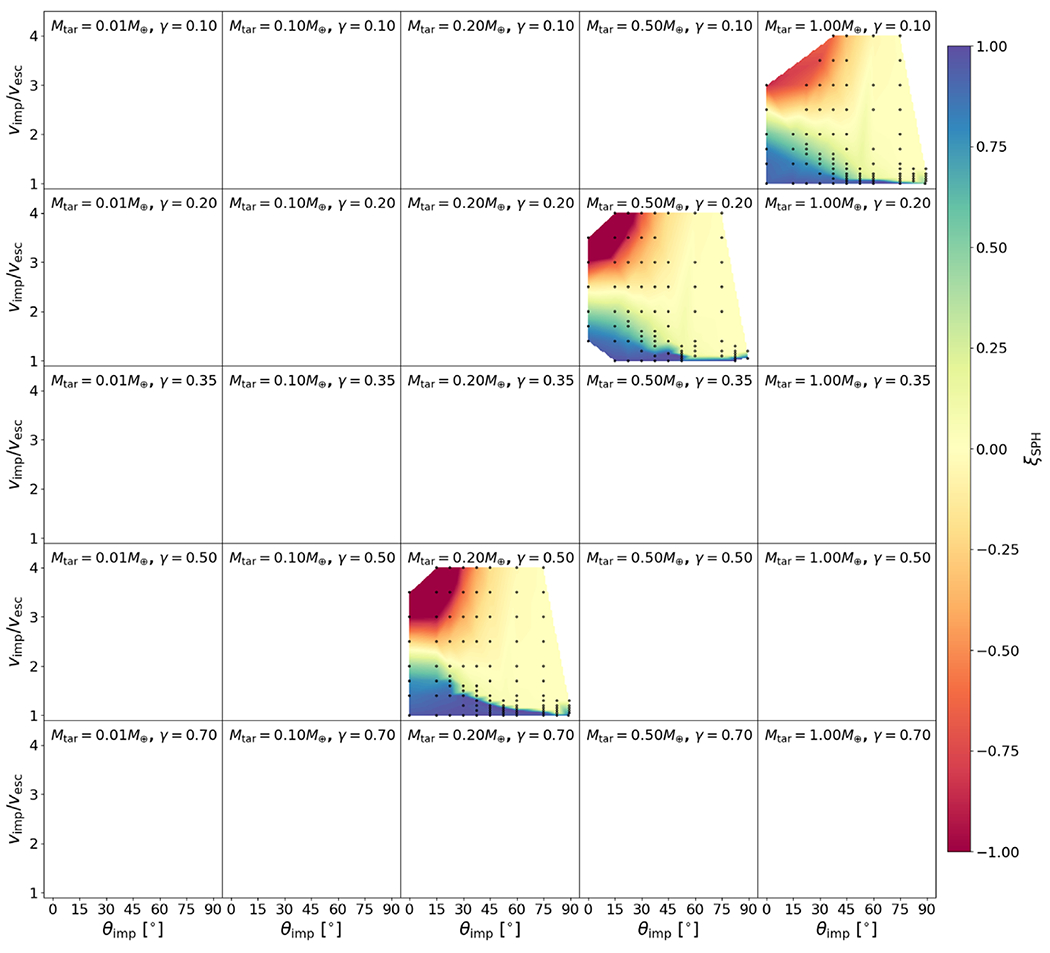
The accretion efficiency for the pure SiO_2_ bodies as a function of impact angle (*θ*_imp_; *x*-axis) and impact velocity normalized by the mutual escape velocity (*ν*_imp_/*ν*_esc_; *y*-axis). Linear interpolation was used to produce the heat map. Each panel represents a unique combination of impactor-to-target mass ratio (*γ*; rows) and target mass (*M*_tar_; columns). Warm colors represent erosive outcomes whereas cool colors represent accretionary outcomes.

**Figure 15.**
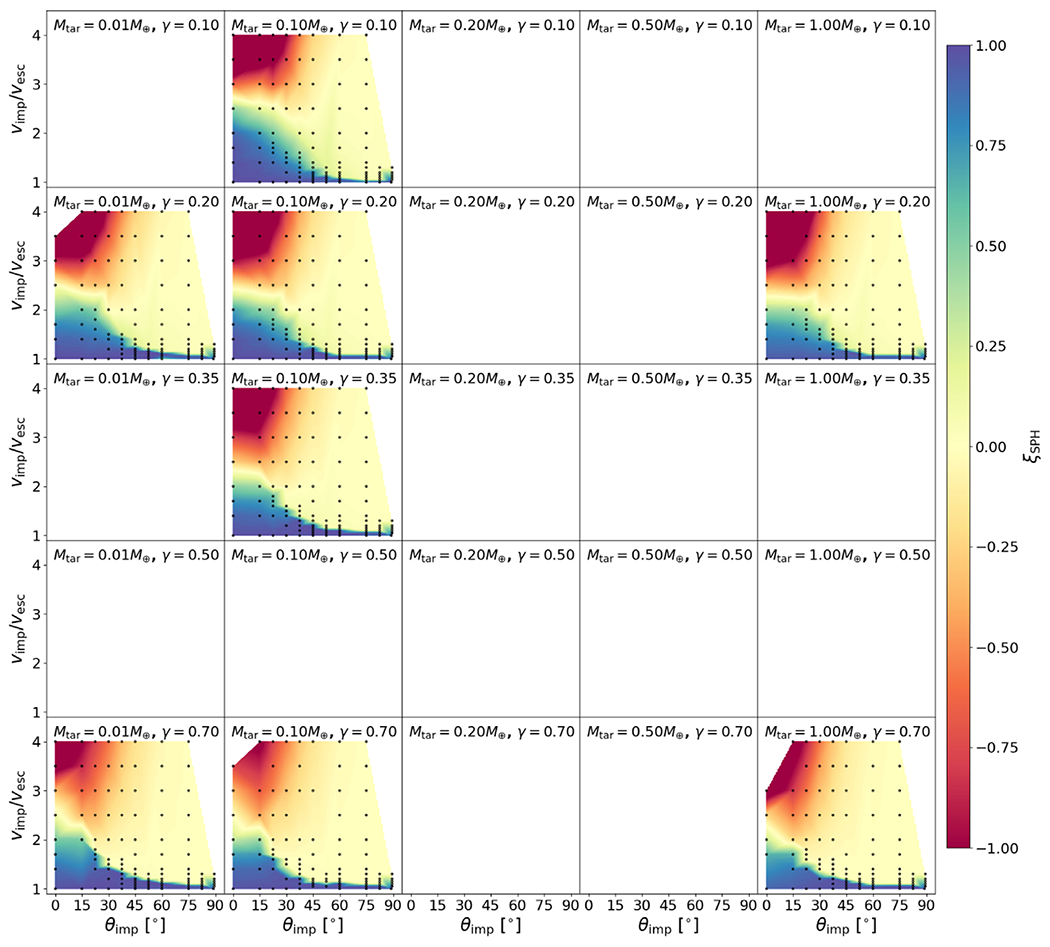
The accretion efficiency for the SiO_2_–Fe bodies. Axes are the same as Figure 14.

**Figure 16.**
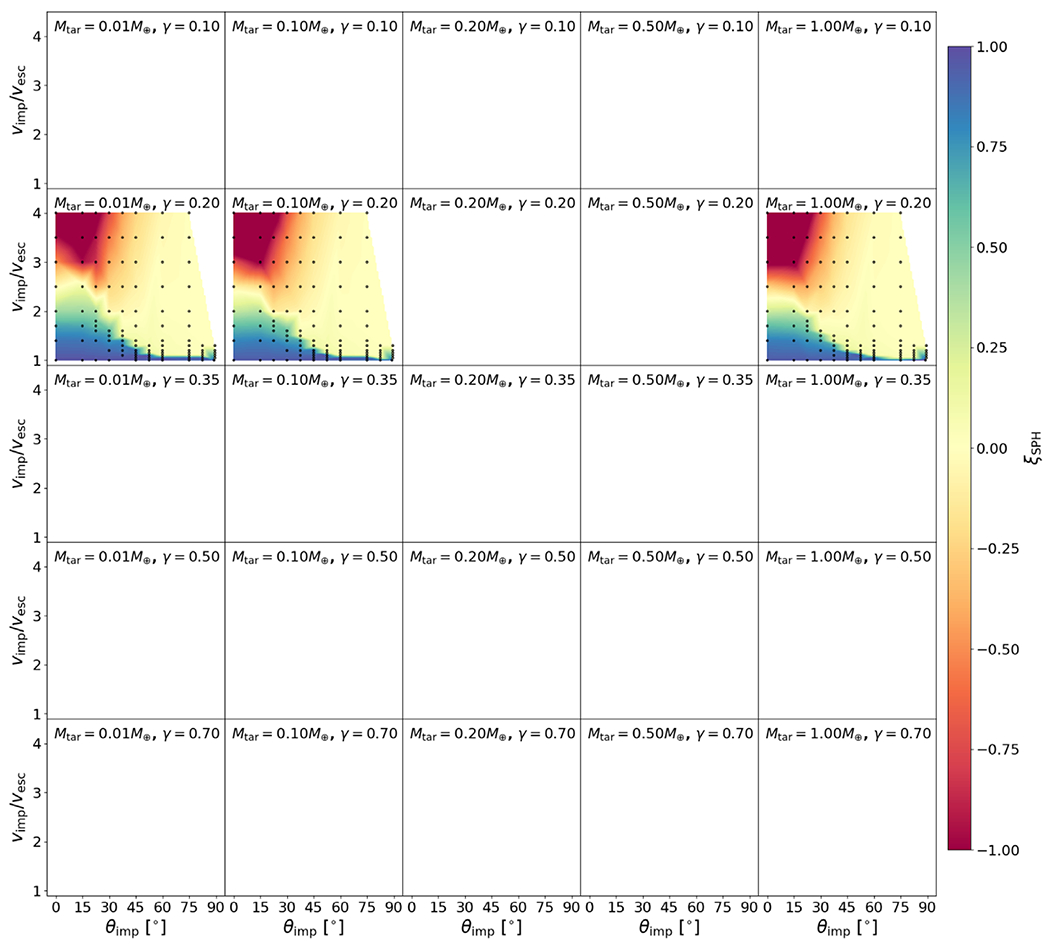
The accretion efficiency for the H_2_O–SiO_2_–Fe bodies. Axes are the same as Figure 14.

**Figure 18.**
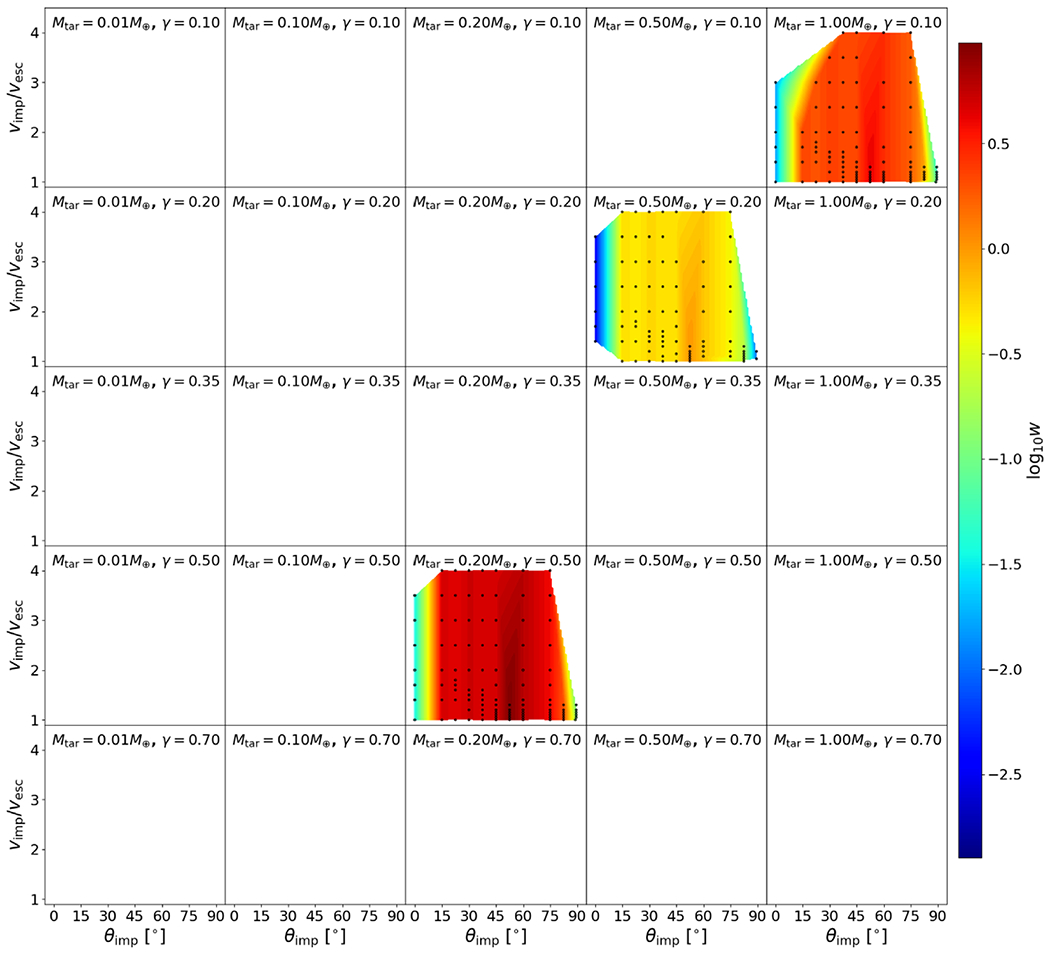
The log of the weights *w_i_* used in the MCMC routine for the pure SiO_2_ bodies. Each panel represents a unique combination of impactor-to-target mass ratio (*γ*; rows) and target mass (*M*_tar_; columns). Cooler colors represent lower weights. (The complete figure set (3 images) is available.)

**Figure 19.**
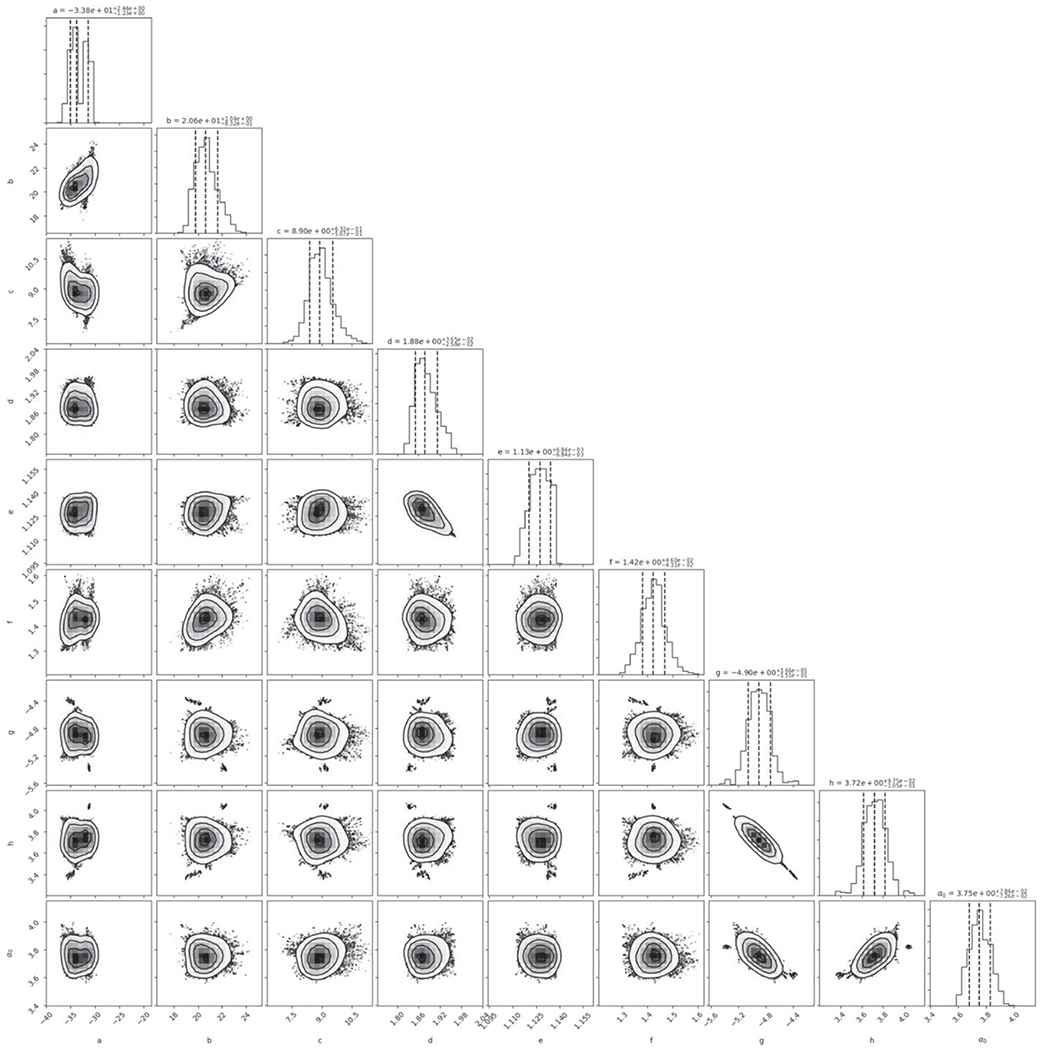
The posterior distributions of the optimized parameters in a series of one-dimensional and two-dimensional histograms (Foreman-Mackey 2016). The scatter density plots in the off-diagonal frames are two-dimensional projections of the posterior distributions; these frames illustrate the covariance of each possible pair of the fitted parameters. Diagonal frames show the marginalized likelihood for each parameter. In each diagonal frame, the 16%, 50%, and 84% quantiles are shown; between the 8% and 84% quantile lies 67% of the likelihood distribution.

**Figure 23.**
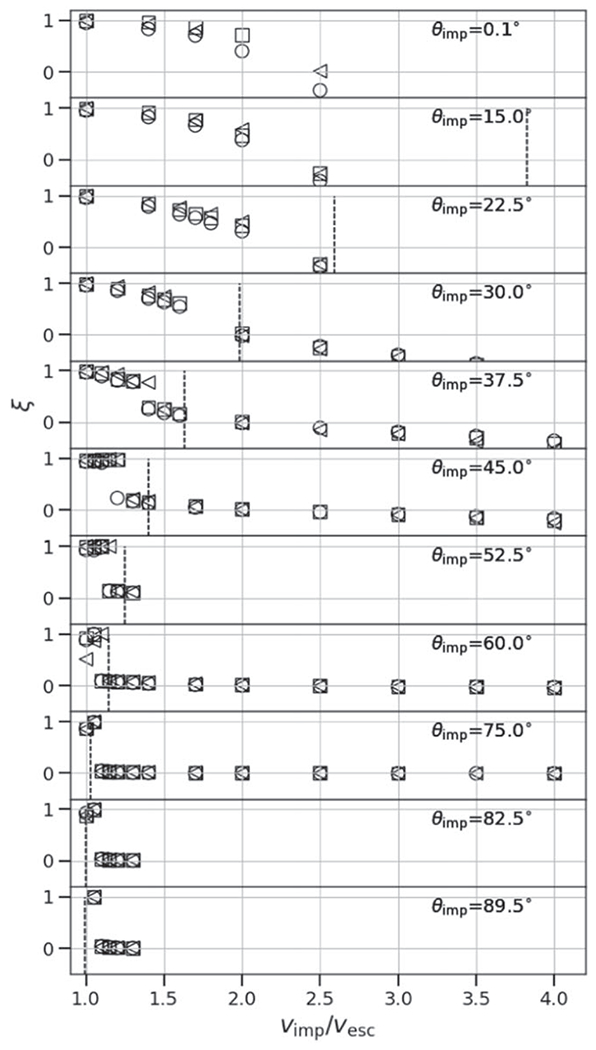
The hit-and-run threshold from Jutzi & Asphaug (2015; dashed line) as a function of impact velocity, compared to the accretion efficiency data from the SiO_2_–Fe simulations in our study. Circle, square, and triangle symbols represent data for *M*_tar_ = 1.0 *M*_⊕_, 0.1 *M*_⊕_, and 0.01 *M*_⊕_, respectively.

**Figure 24.**
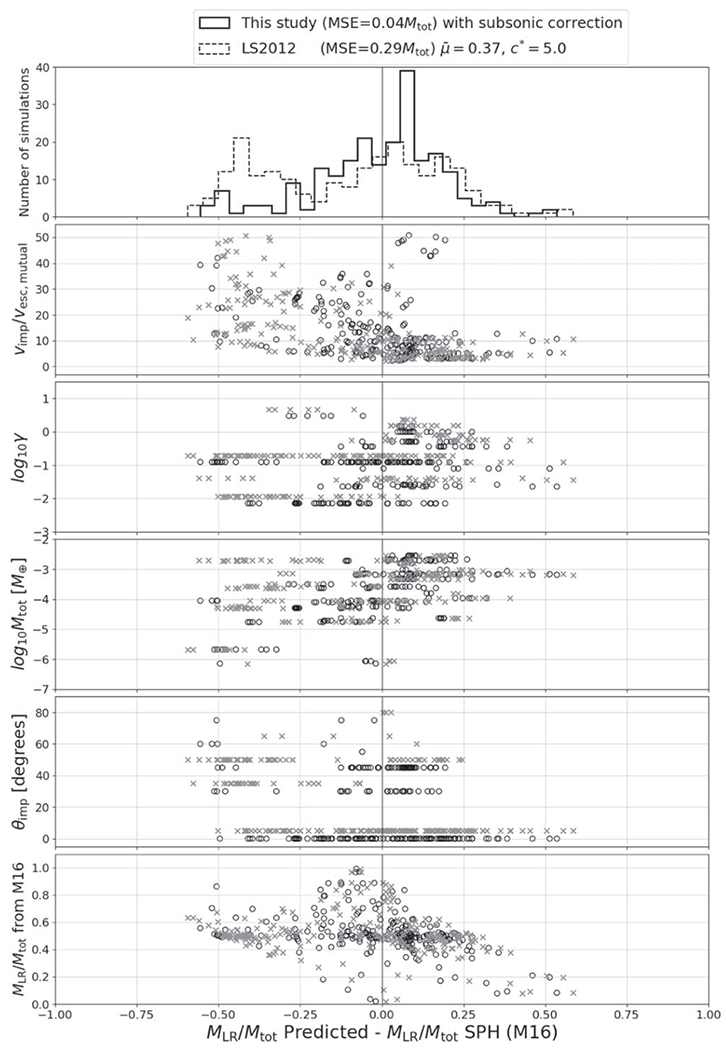
Shown are the residuals of *M*_LR_/*M*_tot_ between giant-impact models and simulation results from Movshovitz et al. (2016, supplementary Table 3 therein). We assumed c* and $\bar{\mu }$ values appropriate for small bodies in the Leinhardt & Stewart (2012) model. The top panel shows the cumulative distribution of residuals. The four consecutive panels present the residuals as a function of impact parameters to examine potential model biases. The ○ and × symbols represent our model and the Leinhardt & Stewart (2012) model, respectively. The last panel presents the residuals as a function of the true value of *M*_LR_/*M*_tot_. Impactor-to-target mass ratio (*γ*) and impact angle (*θ*_imp_) values have been artificially offset by $+0_{.}^{{}^{\circ}}05$ and +5° in the Leinhardt & Stewart (2012) residuals for clarity.

**Figure 25.**
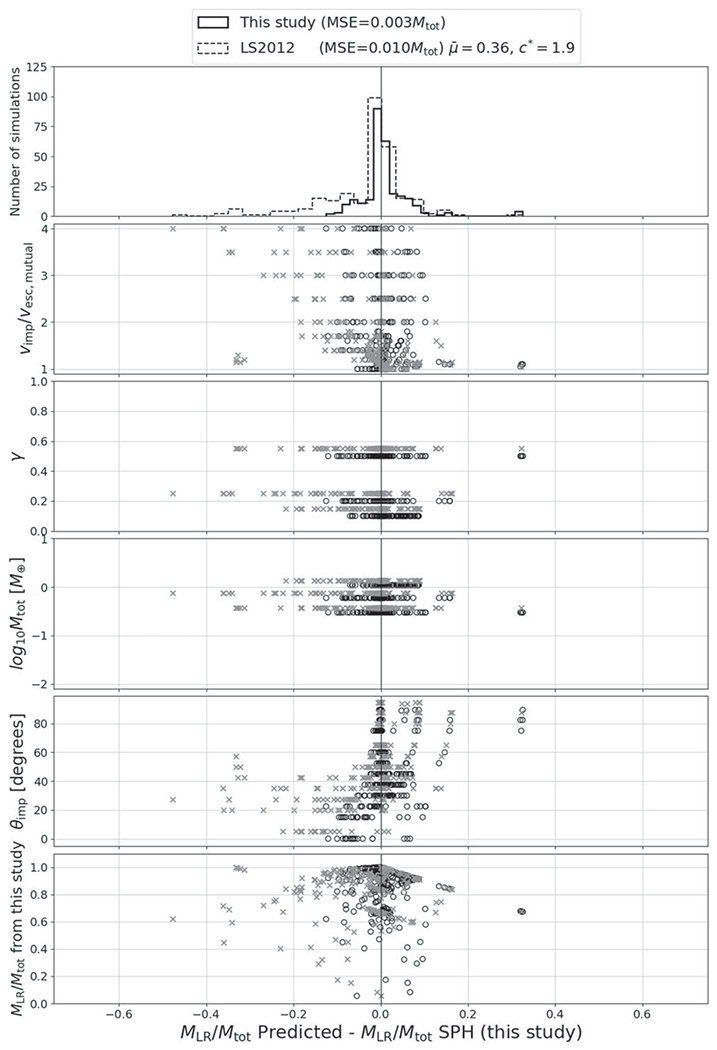
Shown are the residuals of *M*_LR_/*M*_tot_ between giant-impact models and simulation results from this work (pure SiO_2_ bodies). We assumed the $\bar{\mu }$ and c* values appropriate for hydrodynamic bodies in the Leinhardt & Stewart (2012) model. The top panel shows the cumulative distribution of residuals with mean squared error (residual) reported in the legend. The four consecutive panels present the residuals as a function of an impact parameter to examine potential systematic bias in both models. The ○ and × symbols represent our model and the Leinhardt & Stewart (2012) model, respectively. The last panel presents the residuals as a function of the true value of *M*_LR_/*M*_tot_. Impactor-to-target mass ratio (*γ*). total mass (*M*_tot_), and impact angle (*θ*_imp_) values for the Leinhardt & Stewart (2012) model residuals have been artificially offset by +0.05, +0.1 *M*_tot_, and +5° for clarity. (The complete figure set (3 images) is available.)

**Figure 26.**
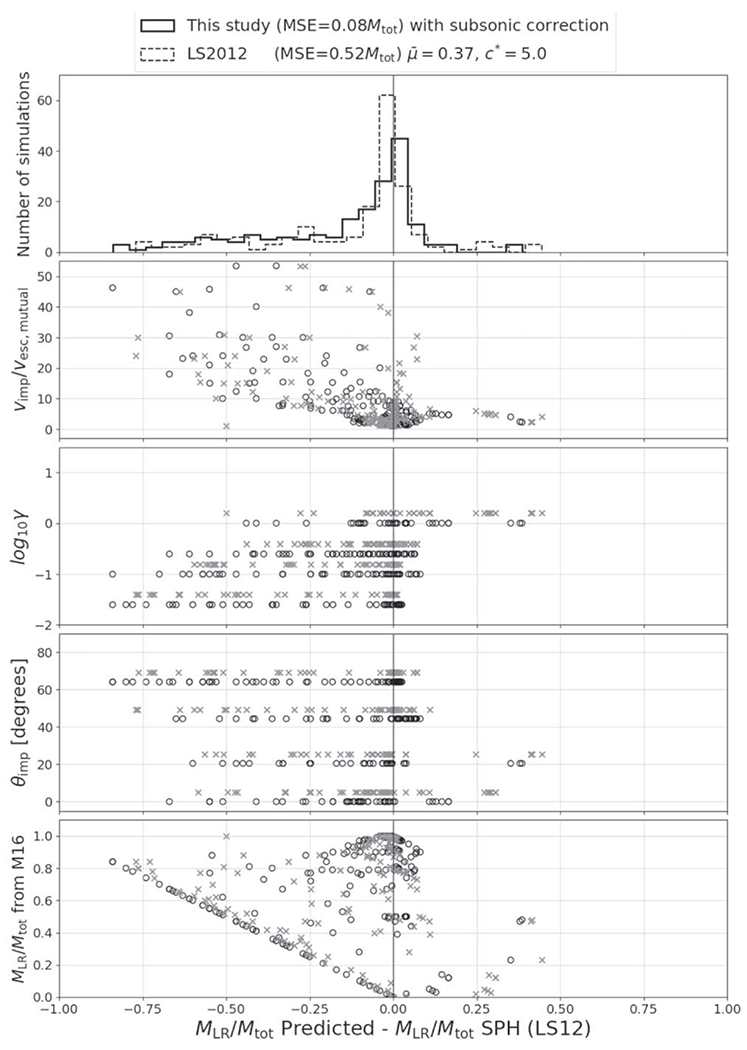
Shown are the residuals of *M*_LR_/*M*_tot_ between giant-impact models and simulation results from Leinhardt & Stewart (2012) (their Table 4). The ○ and × symbols represent our model and the Leinhardt & Stewart (2012) model, respectively. We assumed the $\bar{\mu }$ and c* values appropriate for small bodies in the Leinhardt & Stewart (2012) model. The top panel shows the cumulative distribution of residuals with mean squared error (residual) reported in the legend. The three consecutive panels present the residuals as a function of an impact parameter to examine potential systematic bias in both models. The last panel presents the residuals as a function of the true value of *M*_LR_/*M*_tot_. Impactor-to-target mass ratio (*γ*) and impact angle (*θ*_imp_) values for the Leinhardt & Stewart (2012) model residuals have been artificially offset by $+0_{.}^{{}^{\circ}}05$ and +5° for clarity.

**Table 1:** The Parameters of the SPH Bodies Used in the Simulations Herein, Each with a Unique Identifier in the First Column

				$U_{G}$				
Planet Number	Material	*M*, (*M*_⊕_)	*R*, (*R*_⊕_)^a^	Numerical (erg)^b^	Analytic (erg)	Ratio	Nodes (10^3^)^c^	Specific Entropy, (J kg^−1^ K^−1^)
P01	H_2_O–SiO_2_–Fe	1.002	1.258	2.125 × 10^39^	1.790 × 10^39^	0.842	100	(3.5, 2.8, 1.8) × 10^11^
P02	H_2_O–SiO_2_–Fe	0.100	0.646	4.012 × 10^37^	3.477 × 10^37^	0.866	100	(3.5, 2.8, 1.8) × 10^11^
P03	H_2_O–SiO_2_–Fe	0.010	0.317	8.170 × 10^35^	7.079 × 10^35^	0.866	100	(3.5, 2.8, 1.8) × 10^11^
P04	H_2_O–SiO_2_–Fe	0.200	0.795	1.316 × 10^38^	1.128 × 10^38^	0.857	20	(3.5, 2.8, 1.8) × 10^11^
P05	H_2_O–SiO_2_–Fe	0.020	0.395	2.633 × 10^36^	2.281 × 10^36^	0.866	20	(3.5, 2.8, 1.8) × 10^11^
P06	H_2_O–SiO_2_–Fe	0.002	0.189	5.428 × 10^34^	4.719 × 10^34^	0.869	20	(3.5, 2.8, 1.8) × 10^11^
P12	SiO_2_–Fe	1.002	1.021	2.478 × 10^39^	2.201 × 10^39^	0.888	100	(2.4, 1.8) × 10^11^
P14	SiO_2_–Fe	0.100	0.534	4.625 × 10^37^	4.203 × 10^37^	0.909	100	(2.4, 1.8) × 10^11^
P15	SiO_2_–Fe	0.010	0.257	9.465 × 10^35^	8.703 × 10^35^	0.920	100	(2.4, 1.8) × 10^11^
P16	SiO_2_–Fe	0.700	0.925	1.334 × 10^39^	1.186 × 10^39^	0.890	70	(2.4, 1.8) × 10^11^
P17	SiO_2_–Fe	0.200	0.657	1.524 × 10^38^	1.368 × 10^38^	0.897	20	(2.4, 1.8) × 10^11^
P18	SiO_2_–Fe	0.070	0.479	2.517 × 10^37^	2.292 × 10^37^	0.911	70	(2.4, 1.8) × 10^11^
P19	SiO_2_–Fe	0.020	0.323	3.043 × 10^36^	2.788 × 10^36^	0.916	20	(2.4, 1.8) × 10^11^
P20	SiO_2_–Fe	0.010	0.256	9.482 × 10^35^	8.755 × 10^35^	0.923	10	(2.4, 1.8) × 10^11^
P21	SiO_2_–Fe	0.035	0.385	7.792 × 10^36^	7.128 × 10^36^	0.915	35	(2.4, 1.8) × 10^11^
P22	SiO_2_–Fe	0.007	0.229	5.198 × 10^35^	4.784 × 10^35^	0.920	70	(2.4, 1.8) × 10^11^
P23	SiO_2_–Fe	0.002	0.151	6.433 × 10^34^	5.950 × 10^34^	0.925	20	(2.4, 1.8) × 10^11^
P24	SiO_2_	1.000	1.117	2.116 × 10^39^	2.007 × 10^39^	0.949	250	4.0 × 10^11^
P25	SiO_2_	0.500	0.912	6.451 × 10^38^	6.141 × 10^38^	0.952	250	3.5 × 10^11^
P26	SiO_2_	0.200	0.704	1.310 × 10^38^	1.273 × 10^38^	0.972	100	3.0 × 10^11^
P27	SiO_2_	0.100	0.570	3.974 × 10^37^	3.926 × 10^37^	0.988	50	2.4 × 10^11^
P28	SiO_2_	0.100	0.569	3.975 × 10^37^	3.939 × 10^37^	0.991	25	2.4 × 10^11^

**Notes.** Radii are measured after the bodies are relaxed in SPH for several times the free-fall timescale. The columns under $U_{G}$ represent various values of the gravitational binding energy, from numerical integration or from the analytic solution (Equation (17)). The ratio of the numerical and analytic values demonstrates the degree of density stratification in each of the planets, i.e., smaller ratios mean mass is more centrally concentrated in the body. In this study, we use the numerical value of the two colliding planets to compute the binding energy of the collision using Equation (18). Specific entropy is provided in the last column to allow for the replication of our study under identical thermal conditions. For layered planets, the first number in parentheses corresponds to the entropy of the outermost layer, with deeper materials listed in sequence.

a We compute the radii of relaxed hydrodynamic planets by computing the mean radial position position of the outermost 100 nodes, from the center of mass, and adding the mean of half of their smoothing length, *h*/2, to that value.

b We compared the potential energy reported by sphlatch, which uses the tree-gravity calculation with an opening angle of 0.7, to the shell-integrated value, using 1000 shells, and found agreement to within ~0.5%. With an opening angle of 0, the potential energy had a fractional change of ~10^−4^.

c The true number of nodes varies by less than 10 from the reported values.

**Table 2:** Reported Values of the Fit Parameters Provided by MCMC Optimization

Parameter	Value	Equations
*a*	−33.8	(21)
*b*	20.6	(21)
*c*	8.9	(21)
*d*	1.88	(23)
*e*	1.13	(23)
*f*	1.42	(24)
*g*	10^−4.9^	(27)
*h*	3.72	(27)
^a^*α*_0_	3.75	(27)

**Notes.** The values are the 50th percentile of the posterior distributions of the fit parameters (see Appendix B). The last column lists the equation number in the main manuscript where the parameter is introduced.

a The range of the values directly computed for *α*_0_, by interpolation or extrapolation as appropriate, is ~3.2–5.

**Table 3:** Sample of the Supplementary Table of the Remnant Masses from SPH Simulations of SiO_2_ Bodies

*M*_tar_	*M*_imp_	*R*_tar_	*R*_imp_	*θ*_imp_	*ν*_imp_	*ν*_esc_	$U_{G,\mathrm{num}}$	Λ	*ξ*	*M*_LR_	*M*_2LR_	*M*_esc_
1.195 × 10^27^	5.973 × 10^26^	4.490 × 10^8^	3.636 × 10^8^	15	2.170 × 10^6^	5.424 × 10^5^	2.293 × 10^38^	0.975	−1.836	9.822 × 10^25^	4.701 × 10^23^	1.693 × 10^27^
2.987 × 10^27^	5.975 × 10^26^	5.817 × 10^8^	3.636 × 10^8^	0.1	9.958 × 10^5^	7.113 × 10^5^	8.108 × 10^38^	0.954	0.916	3.535 × 10^27^	0.0	4.986 × 10^25^
1.195 × 10^27^	5.973 × 10^26^	4.490 × 10^8^	3.636 × 10^8^	30	1.627 × 10^6^	5.424 × 10^5^	2.293 × 10^38^	0.975	−0.3935	9.597 × 10^26^	2.360 × 10^24^	8.297 × 10^26^
5.976 × 10^27^	5.976 × 10^26^	7.124 × 10^8^	3.629 × 10^8^	60	9.029 × 10^5^	9.029 × 10^5^	2.377 × 10^39^	0.948	0.9628	6.551 × 10^27^	0.0	2.186 × 10^25^
1.195 × 10^27^	5.973 × 10^26^	4.490 × 10^8^	3.636 × 10^8^	22.5	1.085 × 10^6^	5.424 × 10^5^	2.293 × 10^38^	0.975	0.0821	1.244 × 10^27^	1.365 × 10^26^	4.114 × 10^26^

**Note.** All values are in cgs units, with the exception of unitless parameters (Λ and *ξ*), and angles are in degrees.

(This table is available in its entirety in machine-readable form.)

**Table 4:** Sample of the Supplementary Table of the Remnant Masses from SPH Simulations of SiO_2_–Fe Bodies

*M*_tar_	*M*_imp_	*R*_tar_	*R*_imp_	*θ*_imp_	*ν*_imp_	*ν*_esc_	$U_{G,\mathrm{num}}$	Λ	*ξ*	*M*_LR_	*M*_2LR_	*M*_esc_
5.982 × 10^27^	4.187 × 10^27^	6.512 × 10^8^	5.900 × 10^8^	52.5	1.201 × 10^6^	1.045 × 10^6^	5.155 × 10^39^	0.887	0.019	6.061 × 10^27^	4.077 × 10^27^	2.198 × 10^25^
5.982 × 10^27^	4.187 × 10^27^	6.512 × 10^8^	5.900 × 10^8^	22.5	3.134 × 10^6^	1.045 × 10^6^	5.155 × 10^39^	0.887	−0.583	3.539 × 10^27^	1.582 × 10^27^	5.039 × 10^27^
5.978 × 10^26^	2.092 × 10^26^	3.406 × 10^8^	2.456 × 10^8^	15	1.500 × 10^6^	4.284 × 10^5^	6.826 × 10^37^	0.908	−1.255	3.353 × 10^26^	0.0	4.715 × 10^26^
5.982 × 10^27^	4.187 × 10^27^	6.512 × 10^8^	5.900 × 10^8^	45	1.358 × 10^6^	1.045 × 10^6^	5.155 × 10^39^	0.887	0.0165	6.051 × 10^27^	4.034 × 10^27^	7.613 × 10^25^
5.982 × 10^27^	4.187 × 10^27^	6.512 × 10^8^	5.900 × 10^8^	60	3.657 × 10^6^	1.045 × 10^6^	5.155 × 10^39^	0.887	−0.0177	5.907 × 10^27^	4.082 × 10^27^	1.713 × 10^26^

**Note.** Columns and units are identical to Table 3.

(This table is available in its entirety in machine-readable form.)

**Table 5:** Sample of the Supplementary Table of the Remnant Masses from SPH Simulations of H_2_O–SiO_2_–Fe Bodies

*M*_tar_	*M*_imp_	*R*_tar_	*R*_imp_	*θ*_imp_	*ν*_imp_	*ν*_esc_	$U_{G,\mathrm{num}}$	Λ	*ξ*	*M*_LR_	*M*_2LR_	*M*_esc_
5.978 × 10^26^	1.196 × 10^26^	4.120 × 10^8^	2.519 × 10^8^	45	1.139 × 10^6^	3.795 × 10^5^	4.992 × 10^37^	0.864	−0.1246	5.829 × 10^26^	7.629 × 10^25^	5.831 × 10^25^
5.974 × 10^25^	1.195 × 10^25^	2.022 × 10^08^	1.205 × 10^8^	52.5	2.238 × 10^5^	1.721 × 10^5^	1.019 × 10^36^	0.865	0.0825	6.072 × 10^25^	1.053 × 10^25^	3.879 × 10^23^
5.978 × 10^26^	1.196 × 10^26^	4.120 × 10^8^	2.519 × 10^8^	60	1.518 × 10^6^	3.795 × 10^5^	4.992 × 10^37^	0.864	−0.0450	5.924 × 10^26^	1.056 × 10^26^	1.945 × 10^25^
5.974 × 10^25^	1.195 × 10^25^	2.022 × 10^8^	1.205 × 10^8^	45	1.980 × 10^5^	1.721 × 10^5^	1.019 × 10^36^	0.865	0.868	7.010 × 10^25^	0.0	1.540 × 10^24^
5.974 × 10^25^	1.195 × 10^25^	2.022 × 10^8^	1.205 × 10^8^	89.5	1.807 × 10^5^	1.721 × 10^5^	1.019 × 10^36^	0.865	0.0652	6.052 × 10^25^	1.112 × 10^25^	7.928 × 10^21^

**Note.** Columns and units are identical to Table 3.

(This table is available in its entirety in machine-readable form.)

## Footnotes

5 A strict boundary would be twice the orbital velocity to account for perfectly in-plane retrograde orbits; however, this is exceedingly unlikely. For most systems, the Keplerian boundaries are rather conservative as collision velocities are on the order of ~*eν*_keplerian_ and thus often a small fraction of the Keplerian velocity.

6 This sound speed normalization should be used as a rough guideline as sound speed is dependent on temperature, pressure, and phase. In SPH simulations, the manifestation of the transition would be dependent on the accuracy of the equation of state. For comparison, at 0 °C and 1 atm ANEOS provides *c*_s_ = 1.49 km s^−1^ and 6.29 km s^−1^ for water and forsterite, respectively.

7 In fact, the largest embryo–embryo impact velocity in those simulations is somewhat lower than 3*ν*_esc_; however, as there are very few high-velocity collisions (poor statistics), we use 3*ν*_esc_ as a conservative estimate.

8 The impactor is composed of ~*γ* 10^5^ nodes.

9 Asphaug (2010) describes the differences in resolving power of granular codes (e.g., PKDGRAV) and SPH codes.

10 In Chambers (2013), *θ*_graz_ is equal to *θ*_b_ defined in Asphaug (2010).

11 We directly interpolate the values of *α*_0_ where possible and use linear extrapolation with the two highest velocity data points otherwise.

12 Predicting the mass of the runner in the Leinhardt & Stewart (2012) formalism requires computing the interacting mass of the “reverse impact”; however, *M*_interact_ for the reverse impact, as defined in Equation (48) of Leinhardt & Stewart (2012), has units of volume rather than mass. To obtain a mass, we thus multiplied the volume obtained using their Equation (48) by the bulk density of the target.

## Appendix A SPH Simulation Data

In Tables 3–5 we provide samples of the supplementary tables for the remnant masses computed from SPH simulation data as a function of pre-impact conditions. Since accretion efficiency (Equation (19)) is a common tool used to distinguish impact outcomes, we provide a complete set of heat maps in Figures 14–16 across the entire database.

## Appendix B Markov Chain Monte Carlo Analysis

We utilize the MCMC method to minimize the uncertainties between the *M*_LR_ and *M*_run_ predicted by our empirical model developed in Section 7 and the values of *M*_LR_ and *M*_2LR_ in the ~1400 simulations depicted in Figure 3. Specifically, we use the emcee package (Foreman-Mackey et al. 2013) for the Python programming language and implement the affine-invariant ensemble sampler (Goodman & Weare 2010). The model developed utilizes nine optimized parameters, *a, b*, and *c* from Equation (22); *d* and *e* from Equation (23); *f* from Equation (25); and *g, h*, and *α*_0_ from Equation (28). We posited in Section 5.1.1 that the density stratification parameter Λ is adequate for describing the difference in mass distribution between the bodies of different materials. From Figure 4, it is apparent that the database of simulations is imbalanced in terms of the distribution of Λ and *γ*, e.g., there are many more *γ* = 0.2 simulations than *γ* = 0.35 and there are many more Λ ≈ 0.9 simulations than there are Λ ≈ 0.85. Furthermore, the database includes sampling in *θ*_imp_ that is not commensurate with the true probability distribution of impact angles from a randomized direction (*P*(*θ*) = sin(2*θ*); Shoemaker 1962). As such, we maximize a weighted version of the log of the likelihood function in the MCMC routine to optimize the fit parameters,

(43) $$\begin{array}{l} L\left(a,b,\ldots ,\alpha_{0}\right)\\ =-\frac{1}{2}\sum_{i}^{N}w_{i}\left\{\frac{{\left[M_{\text{LR,data,}i}-M_{\text{LR,model,}i}\left(a,b,\ldots ,\alpha_{0}\right)\right]}^{2}}{\sigma_{\text{LR,}i}^{2}}+\ln\left(2\pi \sigma_{\text{LR,}i}^{2}\right)\right\}\\ \;-\frac{1}{2}\sum_{i}^{N}w_{i}\left\{\frac{{\left[M_{\text{2LR,data,}i}-M_{\text{run,model,}i}\left(a,b,\ldots ,\alpha_{0}\right)\right]}^{2}}{\sigma_{2\text{LR,}i}^{2}}+\ln\left(2\pi \sigma_{2\text{LR,}i}^{2}\right)\right\}, \end{array}$$

where the weight, *w_i_*, for any given data point, *ξ*_data,*i*_, in the grid of simulations is computed as the multiplication of several weights constructed to account for the imbalances of the simulation grid,

(44) $$w_{i}=w_{\gamma ,i}\times w_{\theta_{\text{imp,}}i}\times w_{\sin2\theta ,i}\times w_{\Lambda ,i}.$$

The weights *w_γ,i_* and *w_*θ*_imp__* account for the difference in the grid density of *γ* and *θ*_imp_. We divide the width of a band of the parameter, which is 0 < *γ* < 0.15 for the first band of *γ*, by the product of the total number of simulations in the band and the total range of the parameter (1 for *γ* and 90 for *θ*_imp_). The same is performed for *θ*_imp_ because we do not evenly sample *θ*_imp_, e.g., we do not have simulations in the range of 0° *θ*_imp_ < 15°, but we have simulations in less than 15° increments at higher angles. To account for the probability distribution of impact angles, we then multiply this weight by *w*_sin 2*θ,i*_ = sin(2*θ*_imp_). The weight *w*_Λ,*i*_ is required as each set of materials in the database have different values of Λ (see Figure 4). It is computed by taking the inverse of the product of the number of simulations with the same database and the number of databases (three). Effectively, this final weighting factor assumes the database covers three values of Λ, which is generally the case, but this is indeed a simplification. The impact velocity distribution of the database of simulations is weighted toward lower velocities, with approximately 60% of the simulations lying below 1.5*ν*_esc_. The true distribution of impact velocities for embryo–embryo collisions may be even more heavily weighted toward lower velocities, for example, in Chambers (2013), ~95% of the impacts occurring below ~1.6*ν*_esc_ (see Figure 17). We tested rebalancing the weights across the impact velocity parameter and weighting according to the distribution in Figure 17, but find that while the optimized parameters in that case provide tight fits to the low-velocity data, they produce vastly poorer results for high-velocity data, as might be expected. We choose not to implement a velocity-weighting scheme beyond that resulting from the structure of the database, particularly because doing so provides well-balanced residuals across the parameter space, allowing for the wide applicability of the optimized model. We also note that the impact velocity distribution is dependent on the dynamical system and the impact accretion/erosion model used in the *N*-body simulation, and within any system/model is likely to be a function of time and/or radial location. This contrasts with the impact angle distribution, which is theoretically derived and not expected to vary between systems.

The weighting scheme can be qualitatively understood by examining the set of grid-based figures. In Figure 18 and the associated figure set, we show the weights *w_i_* to demonstrate they are commensurate with the intended behavior. For example, they demonstrate that near-head-on (*θ*_imp_ ≈ 0) or highly off-axis *θ*_imp_ ≈ 90° collisions have a lower weight across the entire database, due to accounting for the probability distribution of impact angles, *P*(*θ*_imp_) = sin(2*θ*_imp_). The SiO_2_ data generally show higher weights, due to the fact that there are much fewer simulations for that range of Λ; the SiO_2_–Fe data are most numerous and the H_2_O–SiO_2_–Fe data exist only for *γ* = 0.2 where sampling is greatest, and thus both are weighted less compared to other data sets and *γ* values, respectively.

The MCMC routine was initialized with a number of walkers that was eight times the number of free parameters (72 walkers in this case), and the routine was allowed to iterate for 100,000 steps. The last 5000 steps were used to compute a priori for a “production” run of 20,000 iterations. However, rapid convergence was observed, i.e., the median values of the parameters did not change substantially after 20,000–30,000 iterations in the extensive burn-in stage. We assume constant values of uncertainty, *σ*_LR_ = (0.05/(1 + *γ*))*M*_LR_ and *σ*_2LR_ = (0.05/*γ*)*M*_2LR_, to account for numerical errors in the clump-finding algorithm, which is used to compute the mass of remnants. This choice effectively increases the overall weight of deviations in *M*_LR_ over those in *M*_2LR_, and we consider this a reasonable approach given that the second largest remnant encompasses less mass in the simulation and is thus less dependable. The posterior distribution of the optimized parameters is shown in Figure 19, and the median values are reported in Table 2.

Figure 20 and the associated figure set shows the residuals in *M*_LR_/*M*_tot_ between the MCMC-optimized model and the simulated data. Deviations are generally less than 0.1 *M*_tot_, except in the *γ* = 0.7 data for the SiO_2_–Fe bodies; however, the region of higher deviations in that data is along a thin margin where *ν*_HnR_ is slightly overestimated or underestimated. Figures 21, 22, and their associated figure sets show the residuals in *M*_run_/*M*_imp_ and *M*_esc_/*M*_tot_, respectively, between the MCMC-optimized model and the simulated data. Our model is shown to reproduce the underlying data set to within roughly ±0.1 *M*_tot_ for the largest remnant mass; localized errors can be higher in regions where the transition to hit and run is not exactly predicted (see the lower-right panel of the second figure in the set associated with Figure 20). For the runner mass, our model tends to overestimate the mass of the runner in the runner-disruption regime, i.e., for large velocities and at semi-glancing angles (*θ*_imp_ ≈ 30°). Where the transition to hit and run is not exactly predicted, the mass of the runner is naturally underpredicted, resulting in the slim blue regions at low velocities of Figure 21. In terms of escaping debris mass, the conservation of mass assumption provides accuracy to within roughly ±0.1 *M*_tot_. Locally, however, the residuals may reach higher values.

## Appendix C Angular Momentum as a Hit-and-run Criterion

As we have designed our hit-and-run criteria based on both a geometric threshold and a velocity threshold, it is reasonable to examine whether an angular momentum criterion may singularly encapsulate the behavior of the two. For example, Jutzi & Asphaug (2015) found that the onset of hit and run occurred when the angular momentum of the colliding bodies, *L* = sin(*θ*_imp_)*M*_imp_*ν*_imp_(*R*_imp_ + *R*_tar_), reached ~1.4 times the “reference” angular momentum. This is the angular momentum of the bodies colliding at *ν*_imp_ = *ν*_esc_ and *θ*_imp_ = 45°. As shown in Figure 23, at high angles, the criterion from Jutzi & Asphaug (2015) predicts hit-and-run collisions at ~1*ν*_esc_. For lower angles, their prediction diverges rapidly from our velocity criterion (Equation (23)) and our data. We generally find it to predict hit and run at systematically higher velocities at low angles. It is important to note that the scale, composition, and rheology of the colliding bodies are vastly different, which is a reasonable source of the discrepancy. Nevertheless, at near-head-on collisions, the model of Jutzi & Asphaug (2015) predicts hit and run if the impact velocity is sufficiently high. At these impact energies, the outcome would be catastrophic, not hit and run. Adjusting the prefactor to be lower than 1.4 allows it to fit at intermediate angles, but this would then predict hit and run at velocities less than *ν*_esc_ at high angles. Thus, a single critical angular momentum is not sufficient to predict the onset of hit and run, and additional functionality, beyond adjusting the prefactor, is required.

## Appendix D Comparison to Movshovitz et al. (2016) Data

Movshovitz et al. (2016) provide an independent data set of high-velocity, disruptive collisions that we can utilize to make an independent comparison of the remnant mass estimates. In Figure 24, we compare the residuals between our model and SPH simulation data from Movshovitz et al. (2016), as well as residuals between the model of Leinhardt & Stewart (2012) and the SPH simulation data from Movshovitz et al. (2016). Residuals are shown as a function of impact parameters, with underpredictions lying to the left of the zero line and overpredictions to the right. Importantly, both models are roughly centered near a zero residual; however, there are notable differences and biases. The general spread of residuals is larger in Leinhardt & Stewart (2012; see top panel), with a cluster of underpredictions in dissimilar-sized bodies and off-axis geometries (see the third and fifth panels from the top). Our model tends to overpredict the mass of the larger remnant, potentially due to scale-dependent effects. Our model also shows systematic bias with respect to the total mass, with underpredictions for lower total mass and overpredictions at higher total mass. We do not attempt to compare *M*_run_ predictions to data reported by Movshovitz et al. (2016); the second largest remnant in their high-energy, disruptive collisions is more likely to be representative of a debris fragment, which would be considered part of a size distribution of debris encompassed by *M*_esc_ in our SPH simulations, rather than an intact runner. Overall, we find the mean squared error (MSE) is several times larger in the Leinhardt & Stewart (2012) model (MSE = 0.29 *M*_tot_) than ours (MSE = 0.04 *M*_tot_); adjusting the fit values for the Leinhardt & Stewart (2012) model to the “hydrodynamic body” values ($\bar{\mu }=0.36$ and *c** = 5) produces a similar MSE and introduces a greater degree of bias toward underpredictions. It is also important to note that the residuals are not normalized for the probability distribution of impact angles (*P*(*θ*_imp_) = sin (2*θ*)). When normalizing for this effect, the ratio of the mean squared error (MSE) between the two models grows by a factor of 2–3.

## Appendix E Comparison to Our Data

We also compare our model and that of Leinhardt & Stewart (2012) to our SPH simulation data. In Figure 25 and the associated figure set, we show the residuals for the SiO_2_, SiO_2_–Fe, and H_2_O–SiO_2_–Fe bodies, respectively. Both models are observed to provide a tight fit to zero residual, with minimal systematic overprediction or underprediction and minimal MSE. For the pure SiO_2_ (Λ ≈ 1, homogeneous bodies), where the prediction of the hit-and-run angle is similar in both models, there is minimal systematic bias with respect to impact geometry (fifth panel of Figure 25). Instead, both models tend to show slight underpredictions at low angles and overpredictions at high angle; this is likely due to the shared assumption of both models with respect to the shape of the principal disruption curve (e.g., Equations (5), (11), and (30)). There are some notable biases, particularly in the data sets with stratified planets. The Leinhardt & Stewart (2012) model exhibits increased scatter at low impact velocities (second panel of the second figure in the set) and low-to-mid impact angles (fifth panel), which is likely due to the underestimation of the hit and run criterion from Asphaug (2010). Importantly, for angles larger than about 45°, both models agree and tightly cluster around the zero line, the only exception being the few outliers due to imperfect prediction of the hit-and-run velocity transition. When normalizing for the probability distribution of impact angles, as was similarly done in the previous section, the ratio of the MSE between the two studies grows only by a few tens of percent.

## Appendix F Comparison to Leinhardt & Stewart (2012) Data

In Figure 26, we also compare the residuals of the two models computed from the subsonic collision data in Leinhardt & Stewart (2012; their Table 4). Both models show the mode of the residuals is at zero; however, there is a tail of underestimated values in both cases. The bias toward underestimated remnant mass lies primarily in the impact velocity, indicating that the two models are not predicting the catastrophic and supercatastrophic scenarios. This point is also reflected in the final panel, where the residuals lie on a one-to-one line with the SPH results from Leinhardt & Stewart (2012), indicating the models predict a zero value at those points. A slight bias toward underestimation of remnant masses for small γ is also seen. We generally conclude that both models do not predict with high accuracy the supercatastrophic simulation data from Leinhardt & Stewart (2012) and may require refitting to resolve that regime. In terms of the global accuracy, the MSE in our model is lower; however, the discrepancy decreases slightly when correcting for the probability distribution of impact angles.
